# Generative design-enabled exploration of wireframe DNA origami nanostructures

**DOI:** 10.1093/nar/gkae1268

**Published:** 2024-12-31

**Authors:** Anthony J Vetturini, Jonathan Cagan, Rebecca E Taylor

**Affiliations:** Department of Mechanical Engineering, Carnegie Mellon University, Pittsburgh, PA 15213, USA; Department of Mechanical Engineering, Carnegie Mellon University, Pittsburgh, PA 15213, USA; Department of Mechanical Engineering, Carnegie Mellon University, Pittsburgh, PA 15213, USA; Department of Biomedical Engineering, Carnegie Mellon University, Pittsburgh, PA 15213, USA; Department of Electrical and Computer Engineering, Carnegie Mellon University, Pittsburgh, PA 15213, USA

## Abstract

Recent advances in computer-aided design tools have helped rapidly advance the development of wireframe DNA origami nanostructures. Specifically, automated tools now exist that can convert an input polyhedral mesh into a DNA origami nanostructure, greatly reducing the design difficulty for wireframe DNA origami nanostructures. However, one limitation of these automated tools is that they require a designer to fully conceptualize their intended nanostructure, which may be limited by their own preconceptions. Here, a generative design framework is introduced capable of generating many wireframe DNA origami nanostructures without the need for a predefined mesh. User-defined objectives that guide the generative process are input as either single- or multi-objective optimization problems. A graph grammar is used to both contextualize physical properties of the DNA nanostructure and control the types of generated design features. This framework allows a designer to explore upon and ideate among many generated nanostructures that comply with their own unique constraints. A web-based graphical user interface is provided, allowing users to compare various generated solutions side by side in an interactive environment. Overall, this work illustrates how a constrained generative design framework can be implemented as an assistive tool in exploring design-feature trade-offs of wireframe DNA nanostructures, resulting in novel wireframe nanostructures.

## Graphical Abstract

**Graphical Abstract:**
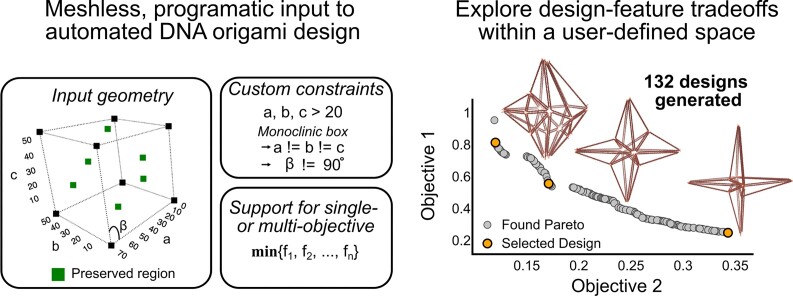

## Introduction

The rapidly maturing field of DNA nanotechnology has facilitated new research avenues in a variety of fields, such as tunable DNA-mediated crystals (1,2) and drug delivery vehicles (3), in part due to the programmability of Watson–Crick–Franklin base pairing (4). A subfield of DNA nanotechnology is DNA origami, a self-assembly process wherein an excess of short, synthetic ‘staple’ oligonucleotides control the folding process of a long, single-stranded DNA ‘scaffold’ into a predefined shape (5). A variety of computer-aided design (CAD) software tools have facilitated the rapid growth of novel DNA origami structures (6,7). However, a common issue with early CAD software for DNA origami design, such as the popular caDNAno (8), is that they can be difficult to use and often require a lengthy, iterative process as a designer must define a nanostructure nucleobase by nucleobase. Furthermore, designing wireframe DNA origami structures, which can be seen as more material efficient and allow for larger nanostructures to be built from a given scaffold length (9), using caDNAno is difficult as it was largely developed for lattice-based DNA origami architectures. Following caDNAno, automated tools such as vHelix (10) and DAEDALUS (11) were introduced, establishing an automated design paradigm that allowed for rapid creation of wireframe DNA origami. Given an input 2D or 3D polyhedral mesh, automated tools will generate staple sequences for a given scaffold to self-assemble the target DNA origami nanostructure, for which the edges of the mesh are represented by single-helix bundle (9,10), double-helix bundle (10–13) (2HB) or six-helix bundle (6HB) edges (14,15). Robust formation of the resulting nanostructures has also been shown experimentally for these automated algorithms. Given the ease and utility of these automated processes, the key limitation for the design of wireframe DNA origami—particularly novel wireframe nanostructures—is now the challenge of creating polyhedral mesh files.

At present, two primary barriers limit the design of custom meshes for wireframe origami nanostructures. First, the complexity of the mesh creation process must be reduced. At present, the creation of an input mesh is not straightforward as many standard CAD software tools will automatically triangulate a mesh. This poses a design barrier as the automatic triangulation may enforce features in a mesh that a designer does not intend, and they must then find alternative methodologies to create their intended mesh. Second, DNA origami designers must rely on their own experiences and expertise to track relevant design features in pursuit of creating what they *believe* is an optimal nanostructure. Any sort of manual optimally directed mesh design process would be especially difficult given that the DNA origami design space is notoriously large and complex (16,17). One way to address both barriers is by using a constrained generative design framework.

Generative design is capable of broadly exploring and producing solutions to a design space. The output of a generative design tool can vary greatly, ranging from a singular, complete (or optimal) solution to generating a set of many nearly optimal solutions to a problem (18). Some generative frameworks are developed through a rules-based approach (19–21) to ensure that only specific design states are sampled given the constraints of the system, whereas other frameworks may use a data-driven model (22,23) to more broadly sample a space. However, current data-driven generative models tend to require large amounts of simulation or physical data, which is a notable limitation of the DNA origami design space (24). Employing generative design within a constrained framework allows a designer to experiment within a design space, and can even help inspire the creation of more complicated designs (25). Historically, generative design has seen adoption in architecture (20,26) and topology optimization (25,27), but has recently seen implementations involving molecular (23,28) and metamaterial (22) design.

Previously, a shape annealing approach to generative design has been applied to DNA origami. Shape annealing (29) uses shape grammars (30) to define a design space and simulated annealing (31) to search the resultant space. This approach introduced a computationally inexpensive, optimization-driven approach to generate a scaffold routing that either coated or filled an input mesh file (17,19). However, this application of shape annealing is only concerned with the routing of the DNA origami scaffold, and in the demonstrated application, the general shape of the generated solution resembles the input mesh. Although this approach shows that optimization can potentially guide the scaffold routing feature of DNA origami, it still requires an input mesh that a designer imagines, whereas many unimagined structures that still fulfill the designer-specific requirements may exist as more desirable solutions. To this end, we present a generative design framework that only requires a minimal set of input conditions such that a predefined mesh is not required. The framework contains generative processes for both single- and multi-objective problems and supports customizable design constraints through a package API, giving a DNA origami designer great control over defining the space in which wireframe nanostructures—many previously unimagined—are generated. The results of the generative process can be viewed using a developed graphical user interface (GUI), allowing a designer to interactively ideate and explore upon generated solutions. A pipeline is shown describing how the discussed framework can be used to consider both size and structural features in generated wireframe nanostructures.

## Materials and methods

### Shape annealing as a generative design framework

#### Shape annealing using graph grammars

Shape annealing (29) is a variation of simulated annealing, a stochastic optimization method that statistically approaches a global optimum among many local optima by probabilistically accepting objectively worse solutions early on in the optimization process in an effort to escape local optima (31), that traditionally uses shape grammars (27) to define moves in the optimization space; early work included the design of truss structures [e.g. (20)]. Shape annealing first considers a feasible solution, *s_n_*, and calculates the ‘energy’ of that solution, denoted by ${{E}_{{{s}_n}}}$. This energy term is nonphysical and stems from an analogy to the metallurgical annealing process (31). Here, the energy term is representative of evaluating an objective (or evaluation) function at the given state, *s_n_*. Next, a random, feasible solution, *s*_*n*+1_, is sampled and its energy is evaluated, denoted by ${{E}_{{{s}_{n + 1}}}}$. These energy values are compared using the Metropolis algorithm (32), where in the case of objective minimization, *s*_*n*+1_ replaces *s_n_* if ${{E}_{{{s}_{n + 1}}}} < {{E}_{{{s}_n}}}$. Otherwise, in the case ${{E}_{{{s}_{n + 1}}}} \ge {{E}_{{{s}_n}}}$, an acceptance probability is calculated as


(1) \begin{eqnarray*}{{P}_{{\mathrm{accept}}}} = - {\mathrm{exp}}\left( {\frac{{{{E}_{{{s}_{n + 1}}}} - {{E}_{{{s}_n}}}}}{T}} \right).\end{eqnarray*}

The acceptance probability is then compared with a randomly sampled value in the range (0, 1). If this randomly sampled value is less than *P*_accept_, then *s*_*n*+1_ replaces *s_n_*; otherwise, *s_n_* is not replaced. A key hyperparameter in the acceptance probability (Equation 1) is the temperature, *T*, which effectively controls the likelihood of objectively worse solutions being accepted (see Supplementary Note S3 for details). Generally, early in the optimization process *T* is a larger value, encouraging explorative behavior in the design space to escape local minima. During the optimization process, *T* is gradually decreased through an annealing schedule (33), decreasing the likelihood of objectively worse solutions being selected.

**Figure 1.**
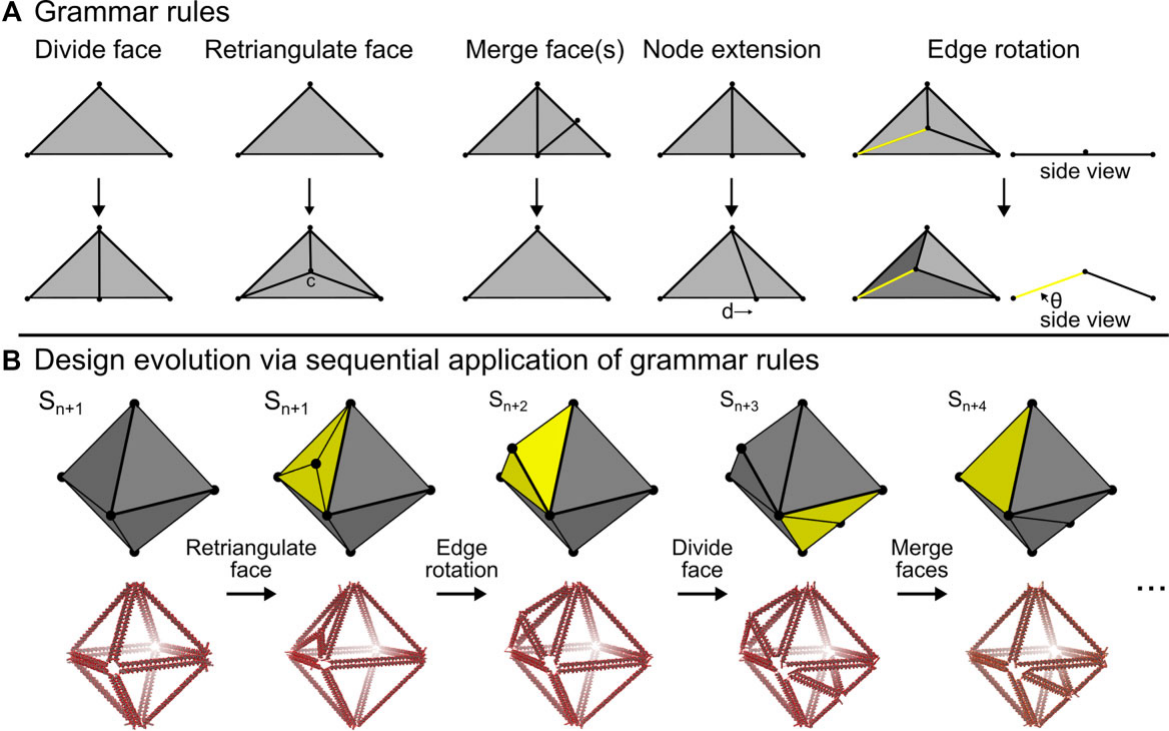
(**A**) The grammar rules in this work involve triangulation of the surface mesh. The distance, *d*, and rotation angle, *θ*, are user-defined values. (**B**) Grammar rules are randomly sampled and applied to evolve a design solution, *s_n_*, during the generative process.

Graph grammar rules (Figure 1A) are randomly selected and applied to a given solution, *s_n_*, until a feasible solution, *s*_*n*+1_, is sampled. A graph grammar is a rules-based approach to generative design, where a sequential application of grammar rules is used to generate a solution (Figure 1B). Previous studies employing optimization-based algorithms typically made design changes at the individual nucleotide level (24,34) or by using shape grammars that leverage the geometry of B-form DNA (17,19). However, the discussed framework is not concerned with the routing of the scaffold, but rather the general shape of the generated nanostructure. Here, graph grammar rules are used to modify the graph that represents the current design solution, *s_n_*, and control the topology of the generated nanostructure. The graph grammar used in this work is similar to a shape grammar (30), where the topology of the current solution is manipulated through a selected rule. Unlike shape grammars, labeled graph information is used to validate a design and ensure that a grammar rule can be applied given the constraints of the problem. The paradigm of the graph grammar rules in this work (Figure 1A) is based on triangulation of the generated mesh, as triangular design features may offer higher structural stability compared to higher order polygons (such as a square) due to a triangle’s ability to distribute experienced forces and maintain its shape (35).

Another benefit to a graph grammar as a generative tool is that it can be used to encode DNA origami-specific context and ensure that only valid design states, *s_n_*, are sampled given the grammar rules of the framework (Figure 1). For example, graph grammars have been previously used to impart valency restrictions onto generated molecular solutions to prevent infeasible molecules from being sampled during the generative process (21). For the grammar rules in this work (Figure 1A), the edge lengths of the surface mesh are representative of a discrete number of nucleotides in a DNA origami design. To consider this DNA origami-specific context, the node extension grammar rule constrains the surface mesh edge lengths to values that are multiples of 0.34 nm, the nucleotide-to-nucleotide rise in B-form DNA that is used in DNA origami. Multiples of 0.34 nm are used so that large enough design changes can be made to adequately search the design space (Supplementary Figures S4 and S5). The value of the extension can also be modified with a ramp element during shape annealing to fine-tune a generated solution (Supplementary Note S4).

#### Initializing a design solution without requiring an input mesh

The underlying data structure of this framework that stores the active design state, *s_n_*, is an undirected, labeled graph whose nodes correspond to vertices of a polyhedral mesh, and whose edges map to the edges of the mesh. A graph is a well-suited data structure as it allows various nodal/edge attributes, such as Cartesian positions in space, to be efficiently tracked and updated throughout the generative process. A bounding box element is used to prescribe maximal values by which to constrain a nanostructure in three-dimensional space.

**Figure 2.**
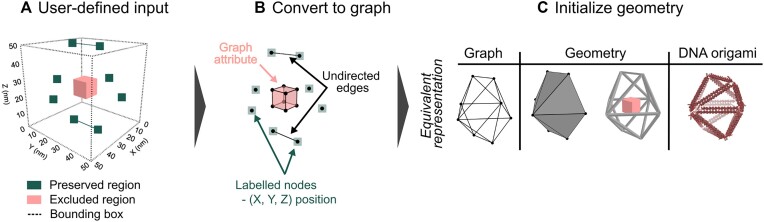
An overview of the input conditions required by this framework. (**A**) The user prescribes a set of preserved and excluded regions. (**B**) These regions are used to initialize the graph that stores the design information in the generative framework. (**C**) The resulting input is triangulated, and the resultant graph nodes and edges are directly representative of a 3D mesh that is also representative of a DNA origami nanostructure.

The input to the generative design framework is a prescribed set of input conditions called *preserved regions* and an optional set of *excluded regions* (Figure 2A). Preserved regions consist of user-defined vertices and/or edges that must exist in a final design; therefore, these geometries will not be removed during the generative process. Preserved regions give a designer a way to enforce a minimal set of input conditions by only defining where material is absolutely required for their own desired application without needing to fully define a mesh. Excluded regions are an optional input and serve to exclude a space from containing any material during the generative process. For example, if a DNA origami designer wishes to have a separate nanoparticle (e.g. protein) located in an origami nanostructure at a predefined location, then an excluded region would prevent DNA from being added within this space so that the framework guarantees that there is available space for the nanoparticle. The vertices of both preserved and excluded regions are prescribed using 3D Cartesian coordinate positions in the form (*x*, *y*, *z*) and these are stored in the graph via node labels (Figure 2B).

The minimal requirement for the input conditions is that the set of preserved regions must be able to be triangulated (Supplementary Note S5), a process that automatically defines an initial set of edges to the design state, resulting in *s*_*n*=1_ from which shape annealing can begin (Figure 2C). This alleviates the need for a designer to manually create a fully defined mesh file as one only needs to define the Cartesian position of a few predefined points or edges. However, this triangulated state must comply with the design constraints of the problem, and if this is not possible, then the generative process cannot begin.

#### Constraining the framework to ensure feasible solutions

**Figure 3.**
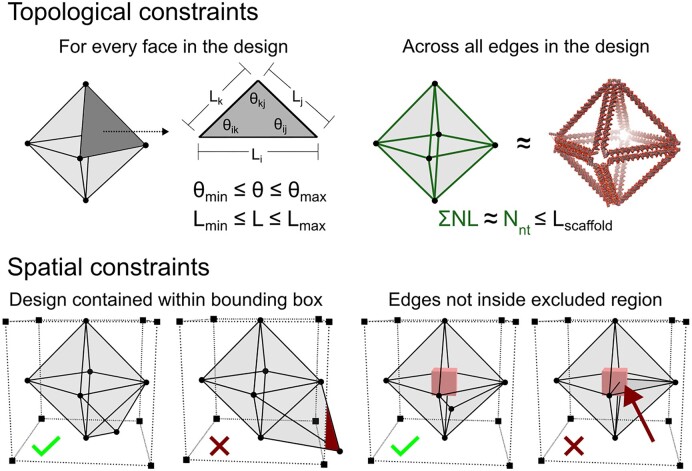
Constraints are used to determine whether a solution is feasible or not. Topological constraints are equality constraints ensuring that a solution meets the requirements for an automated algorithm as well as maintaining a total scaffold length below a set threshold. *N* represents the number of helices per edge, *N*_nt_ represents the estimated number of nucleotides and *L*_scaffold_ represents a user-defined scaffold length in units of nucleotides. Spatial constraints are Boolean and distinguish whether a potential solution is inside the contained bounding box and that no material is inside any prescribed excluded region(s).

Another barrier to creating a mesh for a wireframe DNA origami nanostructure is that there are many constraints that may not be intuitive in a manual design process. For example, automated algorithms found in ATHENA (12) may scale up a nanostructure based on the minimal edge length of the mesh. Therefore, if one were to manually design a mesh with an intended scale in mind, they must track and tune the ratio of edge lengths to achieve their desired scales in the automated algorithm. The scale of a resulting nanostructure determines the total scaffold length needed, and the available scaffold length represents a common constraint for designers. Fortunately, a computational pipeline can easily manage the ratio of edge lengths and ensure compliance with scaffold length requirements. To that end, this framework tracks the relevant state, *s_n_*, features to ensure that all generated solutions comply with the constraints of the problem (Figure 3).

Past these general constraints, a designer may also wish to impose various constraints that are unique to their specific design space. For example, a designer may want to ensure that no two disconnected edges are packed too closely in space to prevent any potential repulsive forces between helix bundles due to the negatively charged backbone of DNA. To enable this level of control, the framework supports an API with which users can define custom functions that determine whether a design is a feasible solution or not. Since the graph nodes are labeled with Cartesian coordinates, a user can define a function relating the positions of the edges to one another. Using this information, a constraint considering a linear repulsive force (36) could be established to limit the design space to nanostructures that only exhibit structural features compliant with a designer’s specific requirements. If the user’s defined repulsive force threshold is exceeded at any generated solution, then that state, *s_n_*, would be deemed infeasible and not considered.

#### Interfacing with external tools for nucleotide-level information

While this framework currently considers a graph representative of a DNA origami design, nucleotide-level information, such as a crossover location within an edge of the mesh, is not truly known until the mesh is converted to a DNA origami design file through the user’s preferred automated algorithm. Software tools such as ATHENA (12) and vHelix (10) can generate DNA origami sequences for input mesh files, whereas other tools such as DNAforge (37) can produce DNA or RNA origami sequences based on the desired automated algorithm. An API has been built and documented that enables the framework to access nucleotide-level detail of a nanostructure for a preferred automated algorithm (see the ‘Code availability’ section for software documentation). While this overview of shape annealing introduces this generative design framework conceptually, the reader is encouraged to see Supplementary Notes S1–S5 for more exhaustive descriptions of its components. Supplementary Figures S1 and S2 show flowcharts for the single- and multi-objective shape annealing algorithms, respectively.

### Materials and origami manufacturing methodology

The required staple sequences for both the 2HB and 6HB nanostructures were produced from the ATHENA (12) software and the corresponding staple sequences are shown in Supplementary Tables S1–S3. The lyophilized DNA oligonucleotides were purchased from Integrated DNA Technologies, Inc. at 25 nmol synthesis. The staples were then rehydrated with nuclease-free water and calibrated to 200 μM. Finally, the staple strands were mixed in equal volumes and used directly in DNA origami folding without any further modification. The M13mp18 scaffold was purchased from Bayou Biolabs at 450 nM concentration. For annealing of the 2HB wireframe, 20 nM of the scaffold and 20 molar equivalents of the corresponding staples were combined into 1× TAE buffer with 12 mM MgCl_2_. For the 6HB wireframe, 20 nM of the scaffold and 20 molar equivalents of the corresponding staples were combined into 1× TAE buffer with 14 mM MgCl_2_ and 100 mM NaCl. All samples used the same annealing schedule over the course of 22 h (95°C for 5 min, 85°C down to 76°C for 5 min/°C, 75°C down to 30°C for 13.75 min/0.5°C, 29°C down to 25°C for 10 min/°C, followed by 15 min at 37°C), as described in (13).

### Experimental characterization protocols

For all DNA origami samples, 20 μl of 20 nM origami solutions were analyzed by electrophoresis in 2% agarose gel in 1× TBE buffer with 12.5 mM MgCl_2_ and 1× SYBR Safe DNA gel stain, run at 70 V for 2 h in ice water and imaged using the Bio-Rad ChemiDoc Imaging System. Samples were purified from excess staples and folding buffer using an Amicon Ultra 0.5 ml spin filter column with a molecular weight cutoff of 100 kDa with no buffer exchange. For negative-stain electron microscopy, 2.5 μl of purified samples were deposited onto freshly glow-discharged copper grids with a support film of graphitized carbon. The sample was side-blotted, washed on a drop of water, blotted again and washed in a 1% solution of uranyl acetate before a final blot. Grids were air-dried for 5 min before imaging in a Tecnai TF20 transmission electron microscope operating at 200 kV (Thermo Fisher Scientific, Waltham, MA, USA). Images were collected on a TVIPS XF416 CMOS camera using the TVIPS Emplified software (TVIPS GmbH, Gilching, Germany). For atomic force microscopy (AFM) characterization, 20 μl of 2 nM unpurified origami solution in TAE with salts matching the sample’s manufacturing methodology was deposited onto a freshly cleaved mica surface and incubated at room temperature in a humidity chamber for 5 min. The samples were washed three times with 100 μl deionized water and blow-dried with nitrogen in between washes. AFM scans of samples preloaded onto a wafer (Park Systems Corp.) were performed using the NX10 AFM system in noncontact mode.

### Coarse-grained molecular dynamics simulation protocol using oxDNA

Coarse-grained molecular dynamics (CG MD) simulations are used to examine the potential structural features of the generated wireframe structures using the oxDNA2 model (38,39). CG MD is used to speak to both thermodynamic and mechanical properties of the DNA nanostructure at longer simulated timescales. A standard procedure is used across all wireframe shapes generated in this work. To conduct a simulation, the generated mesh from this framework is first converted to a DNA origami design file via ATHENA (12). This design is then converted into the oxDNA input format using the ‘CanDo_oxDNA.py’ conversion tool from tacoxDNA (40). A standard minimization–relaxation–simulation procedure is used to explore expected deformation and structural fluctuations in solution. This procedure is further described in Supplementary Note S6.

### Code availability

The generative design framework is an open-source (GPL v3) Python package. The accompanying GUI application is a web browser application and is freely available online. Both tools can be accessed via GitHub (https://github.com/CMU-Integrated-Design-Innovation-Group/Mango). Sample tutorials and framework/API documentation are also provided in the GitHub repository. The initial version of the code and accompanying article data have been deposited into Zenodo and are available at https://doi.org/10.5281/zenodo.13948399.

## Results

### A customizable generative design framework for wireframe DNA origami

#### Prescribing an evaluation function

**Figure 4.**
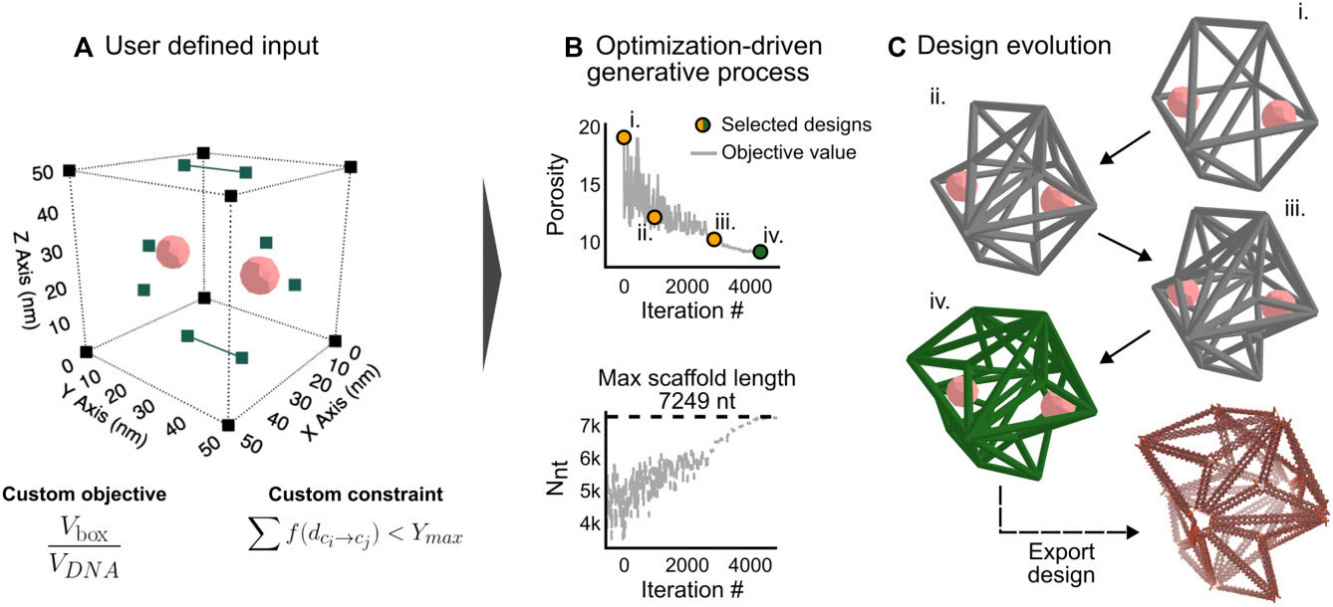
The problem definition for a single-objective shape annealing approach to the described generative design framework. (**A**) The design input considers a bounding box where each dimension is a maximum value of 50 nm. The custom design constraint ensures that no two edges are less than a set distance apart. (**B**) The resultant shape annealing optimization process (top) along with the estimated scaffold length (bottom) required for the design solution at a given iteration. (**C**) A visualization of the design evolution for selected points.

In this framework, an evaluation (or objective) function representing a quantity to be minimized (or maximized) must be specified along with the previously defined input conditions (Figure 4A). This evaluation function is defined by the user and is either minimized or maximized. This function serves to quantify the quality of a generated solution, *s_n_*, in the user’s prescribed space. Overall, the evaluation function corresponds to a specific design goal, such as the utility or manufacturability of the optimal design state. In this first example, a heuristic function is developed for utility, with the aim of exploring ways to increase the volume of a DNA nanostructure within a bounding box. We define a parameter called porosity as


(2) \begin{eqnarray*}{\mathrm{porosity}} = \frac{{{{V}_{{\mathrm{box}}}}}}{{{{V}_{{\mathrm{DNA}}}}}},\end{eqnarray*}

where *V*_box_ is the volume of the bounding box and *V*_DNA_ is the volume of the DNA as represented by a cylindrical model (Supplementary Figure S3). This function will be minimized (approaching a value of 1) as the volume of DNA is increased subject to the constraints discussed in the ‘Materials and methods’ section (Figure 3) as well as a custom linear repulsive force constraint (Figure 4A). This heuristic takes inspiration from cellular studies, where it is suggested that the shape of a wireframe nanostructure may have influence on the innate immunological properties of the design (41). In this example, a designer may want to ideate upon different wireframe shapes conforming to spatial properties of interest. It should be highlighted that due to the modularity of this framework, any function that describes a quality of interest to a designer that can be minimized or maximized can be defined using the package API. This enables a user to explore within their own defined design space using the package documentation (see the ‘Code availability’ section).

#### Framework output

While the output of the generative process is a binary file, custom scripts have been developed and made freely available within the accompanying Python package allowing a user to readily analyze the performance of the optimization process. Generally, the overall performance of the single-objective optimization process can be observed by inspecting the evaluation (objective) function value throughout the optimization process. In objective minimization, it should be expected that the objective function value, in this case porosity, decreases over time to a minimal value. Here, the objective function valuation of the design solution fluctuates greatly early in the optimization process to escape local minima, but over time approaches a value of 9.15 (Figure 4B, top). Due to the constraints of the problem, design solutions exhibiting a lower value of porosity could not be found, namely due to the scaffold length limitation (Figure 4B, bottom). Other than determining performance of the optimization process, a designer can learn more about their design space by considering the solutions at various points during the optimization process. One can discern which structural features are present in the generated nanostructure as opposed to the intermediate, objectively worse, solutions found during the design evolution (Figure 4C and Supplementary Video S1). To enable these comparisons, a web-based GUI is made available, allowing designers to interact with generated designs abiding to their prescribed design space (Supplementary Video S2).

**Figure 5.**
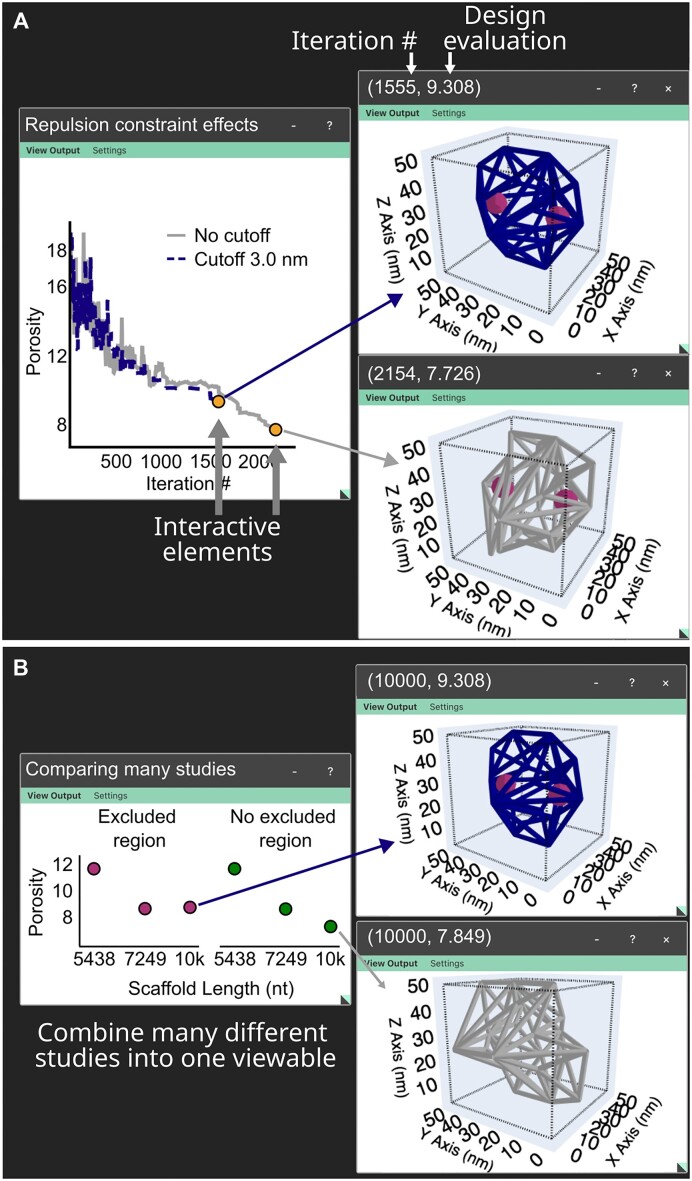
Screenshots of the GUI application. (**A**) Two generative studies are shown where one study considers a repulsive constraint and the other does not use any repulsive cutoff resulting in different generated solutions. (**B**) Multiple studies are combined into one viewable file.

A designer may also be interested in comparing solutions that may have been generated subject to different sets of design constraints to learn more about their prescribed design space. For example, a designer may wish to consider the different types of achievable wireframe geometries with and without the consideration of a linear repulsive force (Figure 5A), or potentially consider how a generated design might look with and without the use of an excluded region (Figure 5B). These problems can all be independently defined and combined into one viewable file, allowing for a direct comparison between generated solutions within an interactive environment. This enables a designer to learn more about their design space as they can visually observe any differences in structural features found in generated solutions abiding to the various constraints.

It should be noted that shape annealing is a stochastic optimizer, and that the tuning of the optimizer hyperparameters is paramount to validating how well converged a generated solution is. The optimizer hyperparameters can be further tuned by the designer to their specific design problem to achieve more optimally directed solutions at the cost of computational time. However, it should be emphasized that the grammar rules of this work are not chosen with the intent of generating the globally optimal design, but rather to inspire ideation and rapidly produce potential solutions abiding to a designer’s specific objective while complying with a set of specified constraints.

### Applying generative design to explore design-feature trade-offs

A potential pitfall of single-objective optimization is that focusing solely on the minimization (or maximization) of a singular objective may converge onto just one portion of the search space. In contrast, multi-objective optimization, which focuses on exposing the Pareto front (or optimal trade-off) of many competing objectives, can lead to the identification of design solutions highlighting the advantages, disadvantages and compromises in a prescribed space (27). This concept is especially important to DNA origami design where the design–property relationships are not yet well understood (16), and optimizing for just one design feature may come at the unintended consequence of other equally important design features. To combat this limitation, the introduced generative design framework also contains a multi-objective optimizer, allowing a DNA origami designer to specify any number of objectives to expose a trade-off between competing performance (evaluation) metrics. While the single-objective optimization process discussed earlier results in a singular design being produced, multi-objective shape annealing (42) produces numerous solutions exposing the Pareto front, or optimal trade-off, of competing objectives. The generated set of designs can then be reviewed by a designer, offering one the ability to explore the varying structural features of many generated nanostructures.

To motivate this example, consider that another potential application of wireframe DNA origami structures is that of DNA origami-mediated superlattices (1,43,44). Promisingly, recent advances have proposed that DNA origami designers have significant freedom in choosing both the internal features and size of building blocks when selecting a wireframe structure used in the self-assembly of crystal lattice structures (45). However, the design of these building blocks is nontrivial in part due to the challenges in wireframe DNA origami design as previously discussed. Here, the generative design framework can be used to produce nanostructures representing the trade-off of designer goals, further illustrating how generative design can be employed to ideate and explore upon potential wireframe nanostructures that satisfy a set of constraints. Furthermore, a variety of automated routing algorithms capable of generating 1–2HB (10), 2HB (12) or 6HB (13) wireframe nanostructures are considered in the generated solutions to conduct a wider search of potential solutions.

Due to the nature of multi-objective optimization, two (or more) competing objectives must be specified. To compete, the minimization (or maximization) of one objective should not directly result in the minimization of the other specified objective(s). The previously defined metric of porosity (Equation 2) considers a design goal of utility, where having more edges within the mesh potentially enables a greater number of modulations that a designer can impart onto the generated nanostructure. Here, multi-objective optimization can be used to find the trade-off between the utility (as defined by porosity) and competing design goals of design uniformity and manufacturability.

The design uniformity can be calculated through a variation index, a measure defined by the standard deviation in the normalized edge lengths of the design:


(3) \begin{eqnarray*}{\mathrm{variation\ index}} = {{{\mathrm{\sigma }}}_{{{L}_{{\mathrm{norm}}}}}}.\end{eqnarray*}

The more uniform the edge lengths are in a design, the lower the variation index will be, thereby minimizing the function. Design uniformity may be an attractive property to DNA origami designers, as many of the experimentally characterized wireframe DNA nanostructures are regular polyhedral or nanostructures with none to modest deviation in the edge lengths (10,12). Alternatively, a measure of manufacturability can be found through the scaffold usage:


(4) \begin{eqnarray*}{\mathrm{scaffold\ usage}} = \frac{{\left| {{{{\bar{L}}}_{{{s}_n}}} - L} \right|}}{L}.\end{eqnarray*}

**Figure 6.**
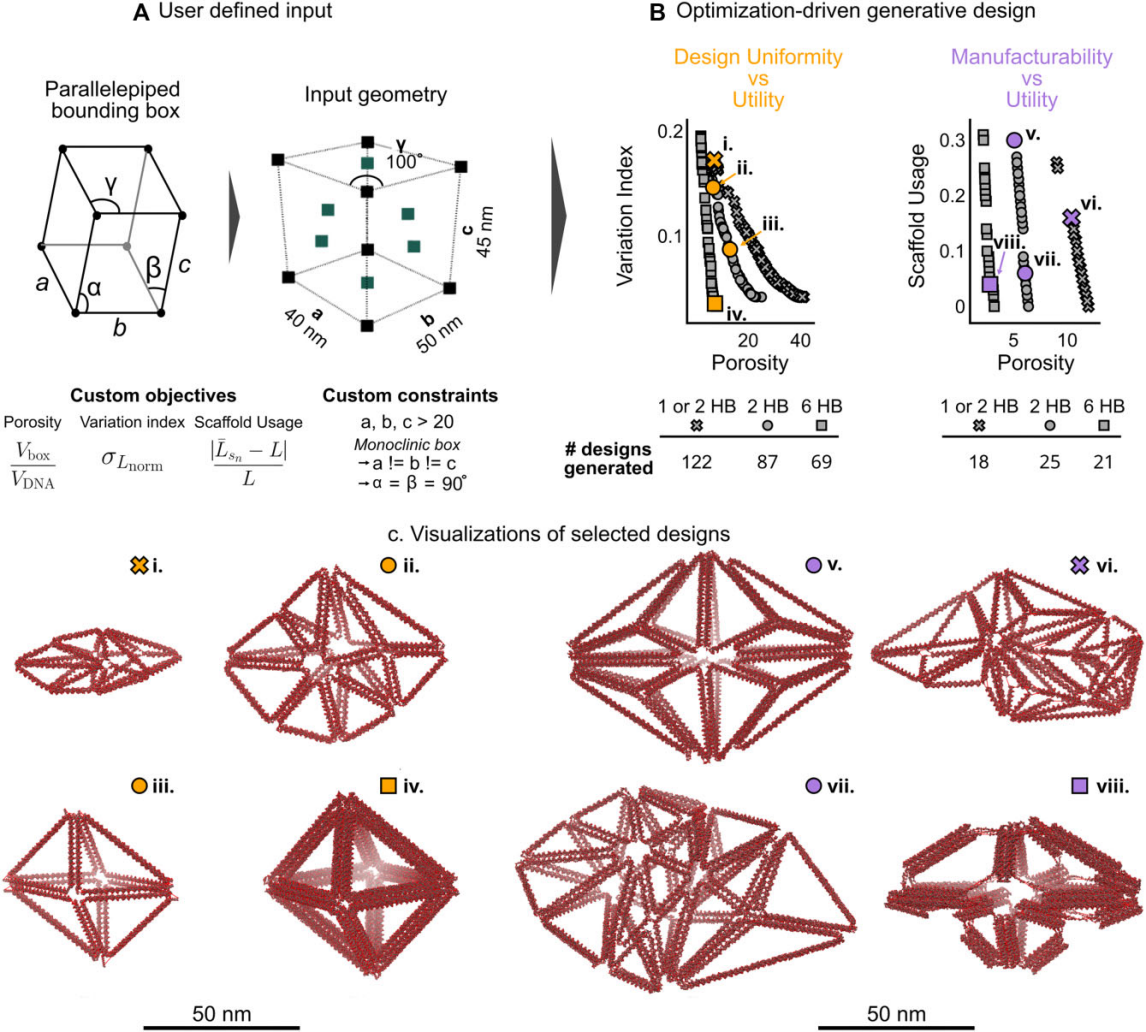
The problem definition for a multi-objective shape annealing approach to the described generative design framework. (**A**) The design input considers a bounding box defined as a parallelepiped where the preserved regions are always held at the midface of each parallelepiped face. (**B**) Two studies showing design uniformity versus utility and manufacturability versus utility are shown. The found Pareto fronts are shown with the number of unique DNA origami designs shown in each table. (**C**) Sample DNA origami nanostructures are shown from each conducted study.

This scaffold usage (Equation 4) considers the scaffold length required for a nanostructure at the current state *s_n_*, denoted by ${{\bar{L}}_{{{s}_n}}}$, compared to a constant scaffold length, denoted by *L*. By considering the scaffold length as a design goal (rather than as a constraint), the generative process can produce solutions that are manufacturable with a specific scaffold. Here, M13mp18 is considered for the scaffold due to its low cost as compared to purchasing or synthesizing custom scaffolds through other means. Using M13mp18 results in a maximum length of *L* = 7249 nucleotides (Equation 4). Overall, as the scaffold usage (Equation 4) is minimized, the design becomes more manufacturable as more of the scaffold is physically incorporated into the generated nanostructure. Finally, these studies are capable of randomly sampling any of the grammar rules (Figure 1A) or modifying the dimensions (*a*, *b*, *c*, *γ*) of the input bounding box (Figure 6A), therefore considering both internal features and size in the generated solutions. It should be noted that while any number of objectives can be specified, multi-objective shape annealing has only been validated with two or three objectives (42).

The resulting process generates a set of designs establishing the found Pareto front for each study, where each data point is a unique design. A wider space is searched when considering the trade-off of utility and design uniformity (Figure 6B, left) as shown by the numbers of generated solutions. There appears to be a higher combinatorial space representing the optimal trade-off of the standard deviation of the edge lengths and the porosity metric. This is likely due to the numerical precision of the variation index, as many of the generated designs with lower variation (<0.1) physically resemble design (iii) or design (iv) (Figure 6C). These structures tend to have one- or two-nucleotide length differences in each of the edge lengths, but the general geometry of the wireframe nanoparticle appears similar (Supplementary Figure S7). Furthermore, although more solutions are generated, many of the generated solutions (Figure 6C, i–iv) are likely not manufacturable with the standard M13mp18 scaffold as the generated nanoparticles incorporate only a much smaller portion of the scaffold. This loss in manufacturability stems from the grammar rule enabling the bounding box volume to change, making the minimization of porosity (Equation 2) less trivial as compared to the single-objective optimization (Figure 4B). Since the bounding box volume can now vary, the scaffold length of the generated solutions does not necessarily drive toward the maximum length constraint (Figure 3) as seen in the single-objective optimization process where the bounding box volume is held constant (Figure 4B, bottom). Conversely, when the trade-off of utility and manufacturability is considered, there is a stark decrease in the number of unique design solutions being generated (Figure 6B, right). The structural features constituting the generated solutions (Figure 6C, v–viii) still vary between designs; however, there is a more direct space that is being searched due to the manufacturability goal. Only designs that are very close to the desired scaffold length (in this case, 7249 nucleotides) are considered within the found Pareto front.

Purely from an optimization perspective, both studies show that 6HB designs are objectively better in both design uniformity and manufacturability versus porosity. The 6HB nanostructures (Figure 6B, square marker) offer lower porosity scores combined with both the variation index and scaffold usage. This can be attributed to the porosity metric which depends on the cross-sectional diameter of the helix bundles. Since 6HB designs have a wider cross-sectional diameter as compared to 1/2HB or 2HB designs, they will naturally exhibit a lower porosity measure. However, there is still justification for conducting these studies from the DNA origami designer perspective. While this multi-objective generative design process considers a variety of design constraints (Figures 3 and 6A), it cannot speak to the simulated or experimentally realized characteristics of the structure. Further, there is a challenge of selecting what the designer may consider the ‘best’ nanostructure from the generated solutions constituting the Pareto front. This challenge stems from the fact that the Pareto front represents the optimal trade-off between competing objectives, and every generated solution is more optimally directed (i.e. ‘better’) in one of the competing objectives. Simulations and physical characterization of these objects may inform a designer’s selection of the ‘best’ solution from the found Pareto front.

**Figure 7.**
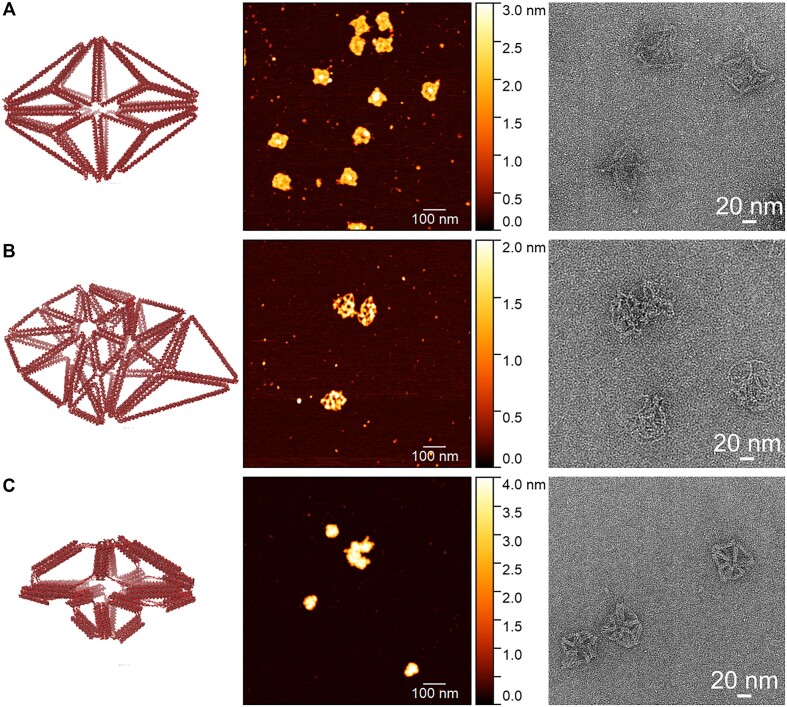
AFM (middle) and negative-stain TEM (right) images of selected DNA origami nanostructures (left). The (**A**) top, (**B**) middle and (**C**) bottom rows correspond to designs (v), (vii) and (viii) from the manufacturability versus utility multi-objective shape annealing process, respectively (Figure 6C).

**Figure 8.**
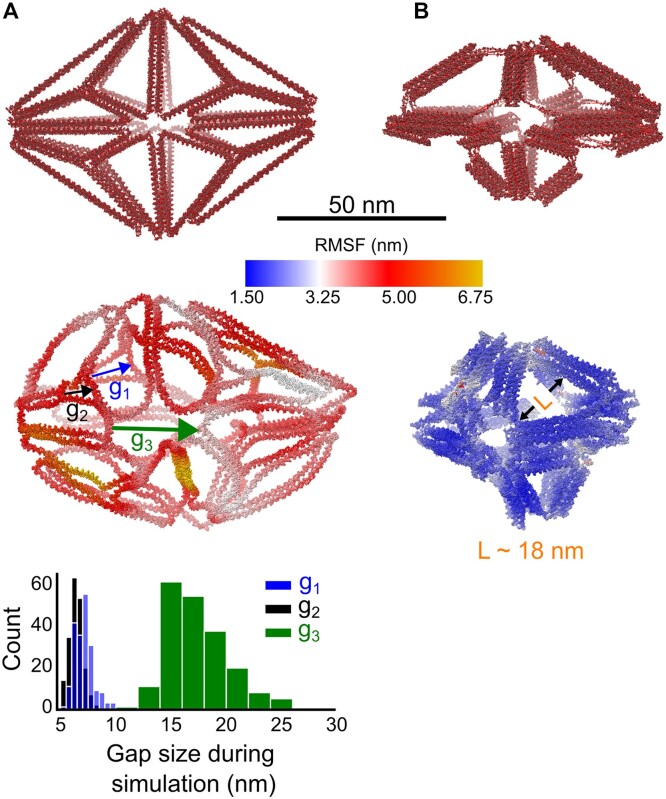
(**A**) Design (v) (Figure 6C) is shown in its as-designed (top) and centroid (middle) configurations. Arrows define which gaps between helix bundles are tracked during the simulation. The resultant histogram shows how large the maximum gap sizes are at these points during the oxDNA simulation (bottom). (**B**) Design (viii) (Figure 6C) is shown in its as-designed (top) and centroid (middle) configurations from the oxDNA simulation trajectory. The maximum length between helix bundles found from the centroid configuration is ∼18 nm.

Both transmission electron microscopy (TEM) and AFM characterization show the formation of nanoparticles for both 2HB designs (Figure 7A and B). AFM shows nanoparticles whose sizes generally match their respective generated design’s size (Supplementary Figure S8). TEM shows that both 2HB structures appear to be structurally soft as their edges appear to fluctuate. Other than experimental characterization, CG MD simulations appear to show an ‘unzipping’ of the 2HB edges when too many edges (helix bundles) are connected to a single vertex (Figure 8A, rightmost distribution). This large gap (measurement described in Supplementary Note S7 and Supplementary Figure S6) effectively alters the global nanostructure dimensions as the helix bundles connected to this vertex in the simulated design are seen to greatly deviate from their designed positions. Conversely, when fewer helix bundles are connected to a given vertex, a similar unzipping at the gap is not observed (Figure 8A, leftmost distributions). This suggests that there may be design-feature coupling between the total number of edges connected at a vertex when generating meshes for wireframe nanostructures with current 2HB automated algorithms. Unlike the 2HB nanoparticles, TEM shows that the 6HB nanostructure appears more structurally rigid compared to the 2HB designs (Figure 7C). However, AFM imaging of the 6HB design appears insufficient to fully characterize this nanostructure, potentially due to the empty space between helix bundles of the simulated nanostructure (Figure 8B), which may not be observed due to the AFM cantilever tip radius. However, the general particle sizes of the 6HB AFM-observed nanostructures generally match the generated design size (Supplementary Figure S9). Gel electrophoresis shows a distinct monomer band for all three structures characterized, although the 6HB nanostructure appears to have the largest amount of aggregation (Supplementary Figure S10).

While this is not intended to serve as an exhaustive analysis of the simulation and characterization of these specific nanostructures, it does exemplify how a designer may consider incorporating generative design into their own design conceptualization workflow in pursuit of a structure that can accommodate their goals. In this example, a designer may continue to iterate with the generative design framework and consider adding further constraints to the maximal number of edges or the variation in the edge lengths connected to any given vertex if the unzipping is interpreted as an inferior structural quality. Alternatively, these simulated and experimental insights can assist a designer in making an informed design decision when considering which design(s) from a Pareto front are the ‘best’ to their own prescribed space.

## Discussion

This work introduces a generative design framework that can create novel wireframe DNA origami nanostructures compliant with a set of user-specific constraints. While many CAD tools in the field require a deeper knowledge of DNA origami design, this tool is poised to help designers explore the different styles of geometries that satisfy their own requirements. By using custom constraints and objectives, one can now generate novel wireframe nanostructures within a designer’s predefined space. Furthermore, we have shown the experimental characterization of nonregular wireframe geometries, supporting how automated design algorithms (10,12,37) can be applied more broadly in the exploration of novel nanostructures. This framework potentially enables other computational tools, such as SNUPI (46) or oxDNA (38), for use as evaluation metrics or custom constraints during a generative process through the package API (see the ‘Materials and methods’ section). To enable these computational tools, external automated algorithms, such as ATHENA (12) or DNAforge (37), must also be considered as currently this framework does not automate the nucleotide-level design. It should also be noted that there is a distinct computational cost with simulations that has been considered (34) to be prohibitively high. One potential avenue to alleviating the computational cost of the simulation is sampling the simulated features of the DNA nanostructure through a pretrained, hybrid data-driven, physics-informed solution such as Deep SNUPI (24). However, as of this writing, the SNUPI software only accepts caDNAno files as input, limiting the application of such a pretrained model. Potentially, a similar hybrid solution can be developed using the more flexible oxDNA/oxRNA format as input, but this necessitates further research and validation due, in part, to the larger computational cost of such CG MD simulations compared to SNUPI.

While the grammar rules in this work consider triangulation of the polyhedral mesh, future work may consider embedding further DNA origami context through a separate or additional set of grammar rules. A consequence of a grammar-based generative design methodology is that generated designs can only exhibit features that are reachable through a sequential application of the grammar rules (Figure 1B). In this work, for example, structural features such as square faces are not reachable due to the design framework enforcing triangular faces. Therefore, one has great ability to tune the grammar rules to generate solutions exhibiting the design features they wish to explore. Future work could also explore shape emergence, a property where new shapes emerge from a set of resulting sub-shapes (47). This could be used to relax the triangular structural feature assumption made in the current grammar set.

This framework also offers an avenue to generating larger datasets of wireframe nanostructures that can capture a variety of features, especially through using multi-objective optimization where a population of designs along a Pareto front is generated. These designs all carry design features that represent the optimal trade-off found in competing objectives. This gives designers an ability to use selected generated designs to potentially learn more about design–property relationships in their own prescribed design space. Overall, this generative design framework offers DNA origami designers a new computational tool for exploring structural features and size-related effects of a variety of DNA origami nanostructures.

## Supplementary Material

gkae1268_Supplemental_Files

## Data Availability

The data underlying this article are available in the article and in its online supplementary material. The generative design framework is an open-source (GPL v3) Python package. The accompanying GUI application is a web browser application and is freely available online. Both tools can be accessed via GitHub (https://github.com/CMU-Integrated-Design-Innovation-Group/Mango). Sample tutorials and framework/API documentation are also provided in the GitHub repository. The initial version of the code and accompanying article data have been deposited into Zenodo and are available at https://doi.org/10.5281/zenodo.13948399.
