# Supplementary material for: Generative design-enabled exploration of wireframe DNA origami nanostructures
Source: Nucleic Acids Res. 2024 Dec 31;53(2):gkae1268. doi: 10.1093/nar/gkae1268 (PMC11754647; doi:10.1093/nar/gkae1268)
Supplement: gkae1268_Supplemental_Files [file gkae1268_supplemental_files.zip › Supplementary Information - Revised_2.pdf]

SUPPLEMENTARY INFORMATION

Generative design-enabled exploration of wireframe DNA origami nanostructures

Anthony J. Vetturini[1], Jonathan Cagan[1,*] and Rebecca E. Taylor[1,2,3,*]

[1] Department of Mechanical Engineering, Carnegie Mellon University, Pittsburgh, PA 15213, USA
[2] Department of Biomedical Engineering, Carnegie Mellon University, Pittsburgh, PA 15213, USA

[3] Department of Electrical and Computer Engineering, Carnegie Mellon University, Pittsburgh, PA 15213, USA

Table of Contents

Supplementary Note 1: Shape annealing description. 3

Supplementary Note 2: Multiobjective shape annealing (MOSA) description 4

Supplementary Note 3: The temperature hyperparameter and the annealing schedule 6

Supplementary Note 4: Grammar ramp element usage and definition 7

Supplementary Note 5: Triangulation of the input preserved regions 8

Supplementary Note 6: Course-grained molecular dynamics simulation protocol 9

Supplementary Note 7: Nucleotide distance tracking in oxDNA simulations 10

Supplementary Figure 1: Shape annealing algorithm as a flow chart 11

Supplementary Figure 2: Multiobjective shape annealing as a flow chart 12

Supplementary Figure 3: DNA as a cylindrical model for porosity objective function 13

Supplementary Figure 4: Ramp effects on node extension distance during the generative process 14

Supplementary Figure 5: A multiobjective optimization process with and without use of a ramp 15

Supplementary Figure 6: Identification of nucleotides for distance tracking 16

Supplementary Figure 7: Sample generated designs showing tradeoff in design uniformity and utility 17

Supplementary Figure 8: AFM imaging and size validation of 2HB nanostructures 18

Supplementary Figure 9: AFM imaging and size validation of 6HB nanostructures 19

Supplementary Figure 10: Gel electrophoresis characterization 20

Supplementary Table 1: DNA Scaffold and Staple Sequences for Design v 21

Supplementary Table 2: DNA Scaffold and Staple Sequences for Design vii 25

Supplementary Table 3: DNA Scaffold and Staple Sequences for Design viii 29

Supplementary References 33

Supplementary Note 1: Shape annealing description.

Shape annealing (1) is the integration of simulated annealing (2) along with shape (or graph) grammars (3). Simulate annealing is a stochastic optimization technique which statistically approaches a global optimum even in complex design spaces. Generally, simulated annealing works by accepting objectively worse solutions early in the optimization process, and over time only accepting objectively better solutions to find a global optimum. This algorithm relies on the Metropolis (4) algorithm which signals that during the optimization process only two design states are ever compared at any given time (i.e., there is no population of designs tracked during the optimization process).

The algorithm starts by considering a feasible design state, denoted s_n. This design state complies with all design constraints of the problem. An energy (or objective function which measures / describes the performance of this state) is evaluated resulting in a measurement E_{s_n}. Following this, the shape annealing algorithm randomly samples an available grammar rule and applies it to the design state, resulting in a new design state s_{n+1}. This new design state is then validated to ensure no design constraints are violated, and if no constraints are violated, then the energy for this design state is calculated resulting in $E_{s_{n+1}}$. If the design constraints are violated in the state s_{n+1}, then this state is immediately discarded and state s_n remains the active feasible state during the optimization process.

In the case of objective minimization, the new design state s_{n+1} will always replace s_n if $E_{s_{n+1}} < E_{s_n}$. If $E_{s_{n+1}} \geqslant E_{s_n}$ then an acceptance probability is calculated as a function of the hyperparameter temperature, denoted T, which is dictated by the annealing schedule discussed in Supplementary Note 3. This acceptance probability can be calculated as:

$$P_{\text{accept}} = \exp\left(-\frac{E_{s_{n+1}} - E_{s_n}}{T}\right). \quad (1$$

Next, a random number in the range (0, 1) is sampled and if this value is less than P_{accept} then the state s_{n+1} is accepted and replaces s_n. Otherwise, the state s_{n+1} is discarded and s_n remains the active design state in which grammar rules are applied. Generally, a larger temperature results in the algorithm accepting objectively worse solutions, and over time as the temperature is cooled, the probability of accepting worse solutions is decreased. For the purposes of this work, the temperature hyperparameter is set as described in Supplementary Note 3.

This process continues for a specified set of hyperparameters by the user. It is recommended to read relevant simulated annealing literature (2, 5, 6) to learn more about how the hyperparameters can further control the optimization process. A full flow chart of the MOSA algorithm is provided in Supplementary Figure 1.

Supplementary Note 2: Multiobjective shape annealing (MOSA) description

The MOSA algorithm (7) developed by Suppapitnarm et al. is a multiobjective optimization algorithm which has been successfully validated against popular genetic algorithms as well as lengthy exhaustive searches whilst maintaining an implementation that is directly compatible and previously used with grammars (8). MOSA maintains an analogy to single objective shape annealing (described in Supplementary Note 1) where a temperature hyperparameter and an annealing schedule are paramount in controlling the conditions of the optimization process. A key difference in MOSA is now any k-number ($k > 1$) objective functions are specified and used to search for a Pareto front of solutions. It should be noted that MOSA has only been tested for 2 or 3 objectives, and if users plan to explore higher dimensional spaces, then further optimizer analysis and considerations must be researched.

This algorithm starts by taking a random walk where grammar rules are randomly sampled and applied to gauge the objective function valuations (hereby called the *performance space* which represents all k-objective function valuations of the design state, s_n). This random walk is used to set an initial temperature value, T_k, for each k-th objective function (5). MOSA uses an archive (analogous to a population) of datapoints that approximates the Pareto front where these points are a mathematically non-dominating set of points measured in the performance space. In the archive, the data points are representative of unique design states. When a new design state is generated through a grammar rule application, it is evaluated for each k-objective function, and those evaluations are compared to the stored archive of designs. If this new design state mathematically dominates any point in the archive, then those states are removed, and the new state is added. If the new state is dominated by any point currently in the archive, then the new state is not added to the archive. Finally, if the new state is neither dominating nor dominated, then it is added to the archive. This process continues based on the hyperparameters of the search.

To avoid local minima, the MOSA algorithm uses an acceptance function to accept objectively worse solutions early in the optimization process. Similar to single objective shape annealing: when the temperature of the objective functions is high, the algorithm is more likely to accept "worse" solutions to hop out of local minima. Over time, the algorithm begins to act more greedily by lowering the temperature hyperparameter. In MOSA, if a design state is not archived, then this is deemed a "worse" move and an acceptance criterion is calculated for all objectives to determine if the design state is used going forward (7). This acceptance criterion is compared using the Metropolis algorithm (4) to determine if the design should be accepted or not. However, it should be noted that even if a worse solution is accepted through the Metropolis algorithm, it is not stored in the archive.

Over time, the MOSA algorithm performs two key operations which influence the search: temperature annealing and a "return to base" strategy. Temperature annealing lowers the temperature hyperparameter for each k-objective, lowering the "worse move" acceptance probability. The return to base changes the active design state to an algorithmically selected

point in the archive at various points throughout the algorithm. Generally, the return to base switches the active design state to the more isolated regions of the Pareto front to encourage a thorough exploration of the performance space. Details for the return to base strategy are described in (7).

The MOSA algorithm runs for a total number of epochs, until a minimal temperature is met through the annealing schedule, or until a prescribed number of iterations is reached. Here, an epoch is defined as every time the temperature hyperparameters are cooled. A full flow chart of the MOSA algorithm is provided in Supplementary Figure 2.

Supplementary Note 3: The temperature hyperparameter and the annealing schedule

As discussed in Supplementary Notes 1 and 2, a temperature hyperparameter is a key hyperparameter used to guide the optimization process in both shape annealing and multiobjective shape annealing. However, the selection of an initial temperature is non-trivial, as the evaluation(s) of the objective function(s) specified can be of varying magnitude(s), which obfuscates what the initial temperature value should be. This work employs a random walk strategy to initialize the temperature as described by White (5). Using this procedure, a random walk of N_{T1} steps are taken, and all objective function valuation(s) are stored. After the walk, the temperature(s) is / are set to the standard deviation of the stored objective function valuation(s). Here, a value of between 500 and 2000 is used for N_{T1} as recommended (7).

The annealing schedule is used to control the temperature hyperparameter during the optimization process. Lowering the temperature results in the optimizer transitioning from explorative to more greedy behavior in the optimization process. By default, the HRSV (9) annealing schedule is used, but this framework also supports the Triki (10) and simple geometric cooling schedules (2) which can affect performance of the optimization process. An example temperature curve is shown in Supplementary Figure 4.

Supplementary Note 4: Grammar ramp element usage and definition

A ramp element can be used to modify the distance and angle of rotation that is used in the triangulation grammar rules shown in Figure 1 of the main article. Specifically, the node extension relies on some distance, d, to move a node and the edge rotation relies on some angle, θ, to rotate an edge about the face it belongs. The ramp element allows for a wider search space because more drastic design alterations can occur earlier in the design process. Over time, as the temperature is cooled (Supplementary Note 3), the ramp will lower the extension (or rotation) value. Because shape annealing acts more greedily due to the lower temperatures, the ramp allows for only smaller design alterations in an effort to fine tune a design(s) to find optimal solution(s).

For example, an extension ramp may vary the distance used by the node extension from 3.4nm to 0.34nm at intervals of 0.34. The value 0.34 is selected due to the presumed axial rise in BDNA which is typically assumed for DNA origami nanostructures. However, it must be noted that this framework is currently only capable of modifying the graph representing the DNA origami design and that these design modifications changes are all approximations. These approximations are validated by converting a design solution into a DNA origami design through an automated scaffold routing algorithm. An example of what a ramp may look like is shown in Supplementary Figure 4.

Supplementary Note 5: Triangulation of the input preserved regions

This framework uses an alpha shape (11) to triangulate the input conditions specified that defines the initial set of edges in a design to begin shape annealing from. An alpha shape is adaptable to either a concave or convex set of input points, enabling the framework to be more generalizable to a wider set of input conditions. This procedure helps alleviate a design barrier regarding mesh design, as a user does not have to fully define a polyhedral mesh prior to the generative process.

The alpha shape results in an initial design state, $s_{n=1}$, from which the constraints of the problem can be validated computationally. If the triangulated solution does not comply with the constraints, the generative process will not begin, and the input criterion (or constraints) must be modified by the user. This helps alleviate a design barrier as a designer would not have to fully define a mesh only to be met with a non-feasible design due to the constraints of the problem. Instead, the designer only needs to specify a few points which can then be automatically triangulated and validated to ensure a design is compliant with the constraints of the problem. This can allow for more rapid prototyping of input conditions that comply with a designer-specific design space.

Supplementary Note 6: Course-grained molecular dynamics simulation protocol

A standard minimization-relaxation-simulation procedure is used in all simulations in this work. The minimization portion is a 2000 time-step process which resolves physical overlaps and issues in the oxDNA file. Following the minimization, the nanostructure is equilibrated through a relaxation process for 10^7 timesteps at 300K using the Langevin thermostat. Following this equilibration, the structure is then simulated for 10^8 timesteps at 300K with a diffusion coefficient of 2.5. The minimization, relaxation, and simulation procedures all use a time step of 0.005. Results are visualized via oxView (12, 13), and analysis of resultant structure is carried out using oxDNA analysis tools (14) where all root mean square fluctuations (RMSF) values were calculated in reference to the centroid structure found by the oxDNA analysis tools. The centroid structure is obtained using the mean structure from the simulation trajectory as the reference. A more comprehensive review of oxDNA parameterization can be read at (15).

Supplementary Note 7: Nucleotide distance tracking in oxDNA simulations

To handle the distance tracking between nucleotides the resultant oxDNA simulation trajectory files are analyzed using oxDNA analysis tools (14). To track the distance, paired nucleotides IDs at each vertex of the design are manually identified using oxView (13). The first paired nucleotide connecting each helix of DNA at the vertices is identified to be used in the distance tracking (Supplementary Figure 7 shows an example). Unpaired nucleotides, representing ssDNA overhangs, are not considered as they will likely fluctuate much more greatly throughout the simulation due to their spring-like nature. Therefore, the first paired nucleotide, representing a more stable point to measure a distance from, at each edge is selected. Finally, a custom script using oxDNA analysis tools measures the maximum distance between all combinations of two tracked nucleotides throughout the simulation trajectory to denote either the maximum gap size (Figure 7 in main article) or the max dimension size (Supplementary Figure 6) in units of nanometers.

Supplementary Figure 1: Shape annealing algorithm as a flow chart

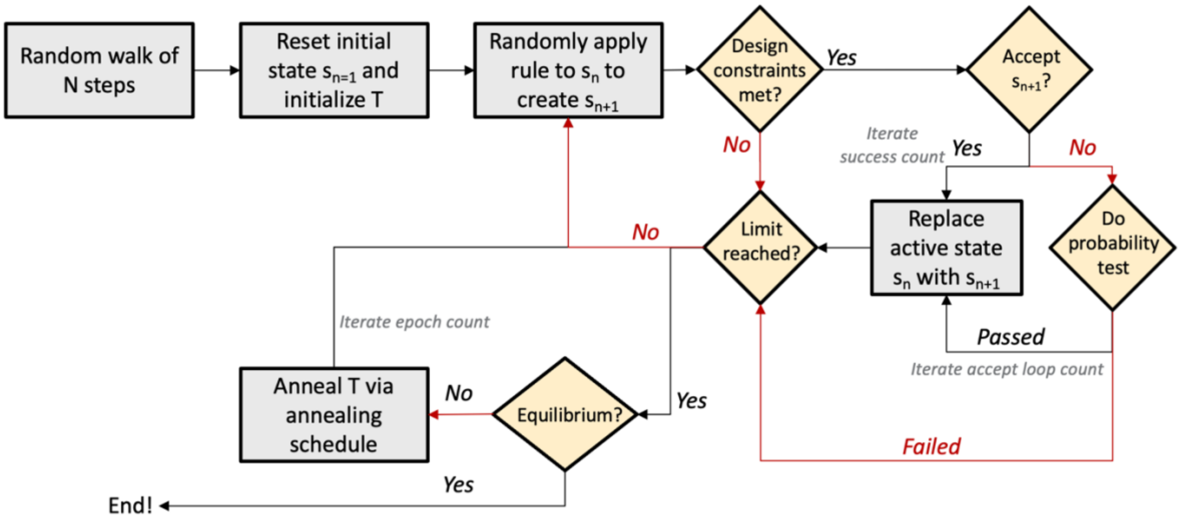

Figure S1: Shape annealing algorithm as a flow chart as described in Supplementary Note 1.

Supplementary Figure 2: Multiobjective shape annealing as a flow chart

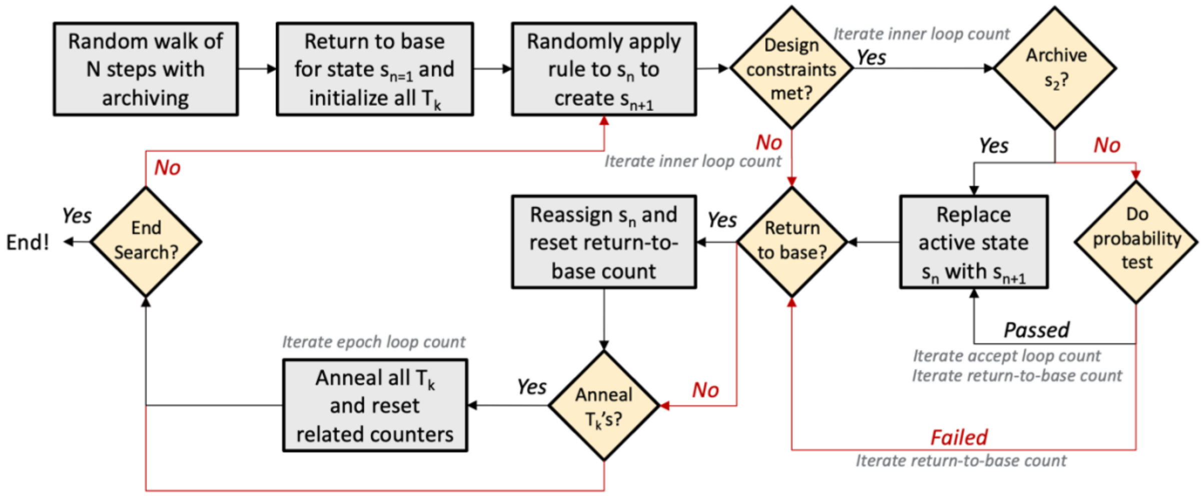

Figure S2: Multiobjective shape annealing algorithm as a flow chart as described in Supplementary Note 2.

Supplementary Figure 3: DNA as a cylindrical model for porosity objective function

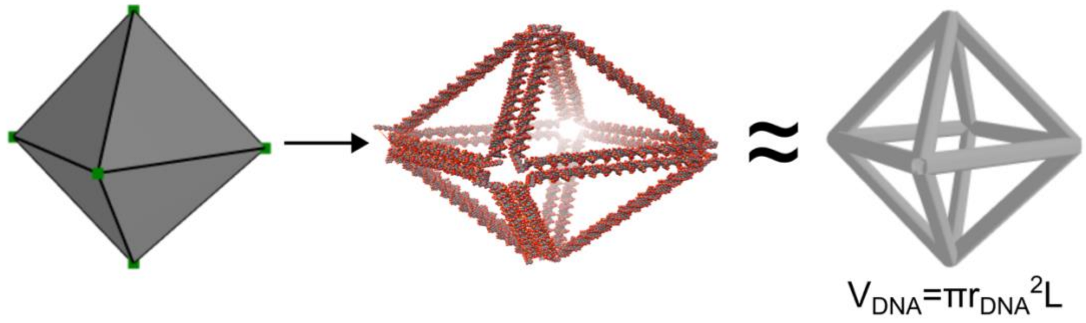

Figure S3: How the volume of a DNA origami edge is estimated where the radius of the DNA helix, r_{DNA}, is equal to the helix-bundle radius (in the case of 2 DNA helices, r~4nm) and L is the length of the edge.

Supplementary Figure 4: Ramp effects on node extension distance during the generative process

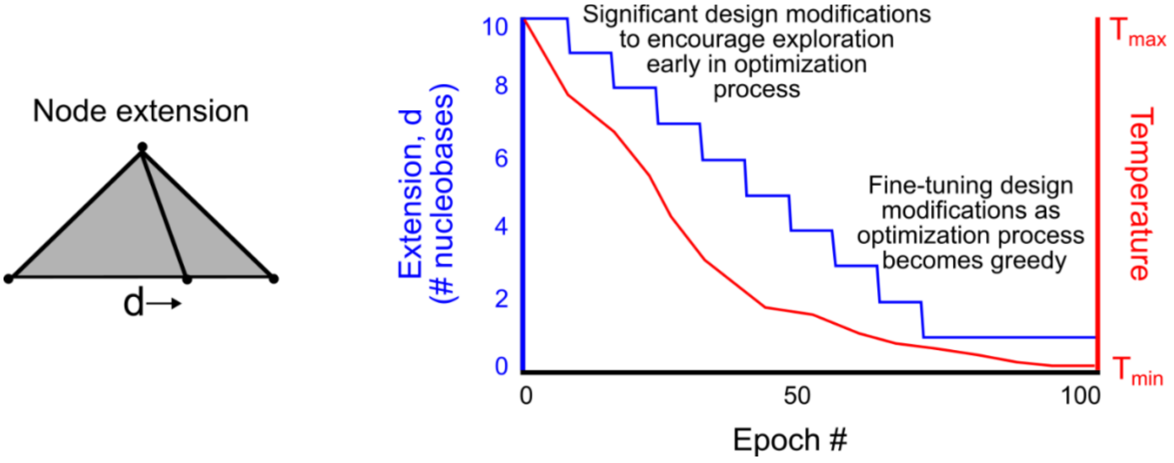

Figure S4: How the node extension value, d, varies during a sample generative process that is running for 100 epochs of length using a ramp. The extension value (blue) is in units of nucleobases instead of nanometers through a scaling factor of 0.34nm (axial rise in B-DNA). The temperature curve (red) is qualitatively shown which controls exploration versus exploitation of the optimization process during the study (Supplementary Note 3).

Supplementary Figure 5: A multiobjective optimization process with and without use of a ramp

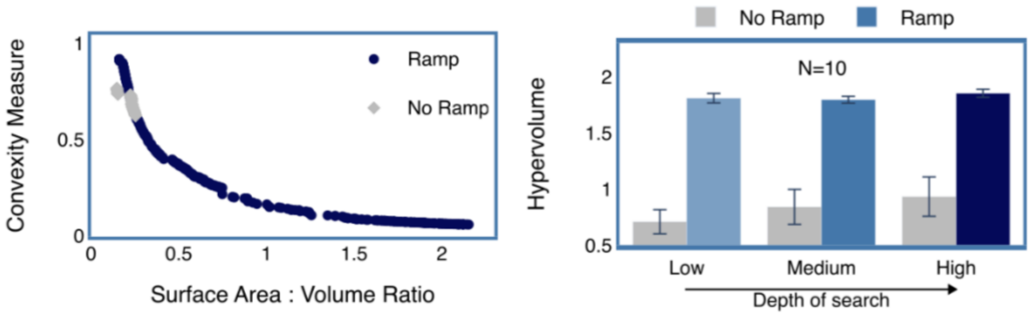

Figure S5. A multiobjective study using the surface area to volume ratio of the design solution polyhedral mesh against a convexity measure defined as the volume of the design solution mesh divided by the volume of the convex hull of the points in the design. Here, the use of a ramp (as described in Supplementary Note 4) allows for wider exploration while using the same optimizer hyperparameters (left). The use of a ramp also allows for consistently better results (measured by the hypervolume (16)) to be obtained across different sets of hyperparameters corresponding to lengthier optimization processes (right).

Supplementary Figure 6: Identification of nucleotides for distance tracking

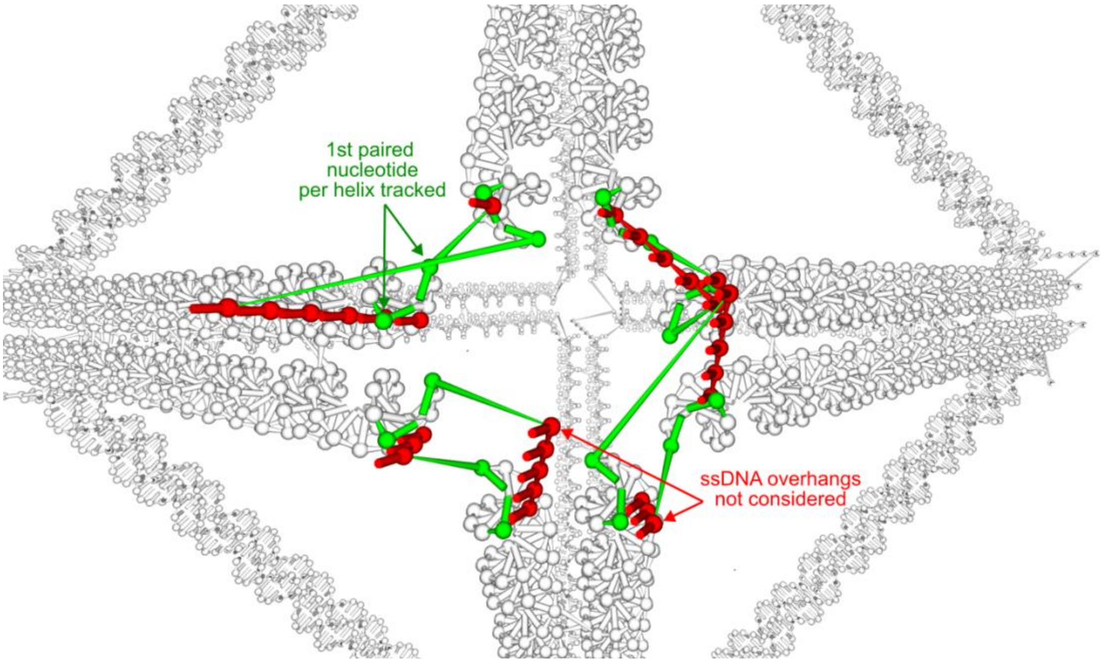

Figure S6: Selection of the first paired nucleotide (colored green) at a vertex for the use in distance tracking for the gap at a vertex (described in Supplementary Note 7) or for the max design dimension in each direction (Supplementary Figure 6). Free nucleotides representing ssDNA overhangs (colored red) are not selected as they fluctuate greatly during simulation. Design visualized in oxView (nucleotide spheres are hidden in the image for clarity) (13).

Supplementary Figure 7: Sample generated designs showing tradeoff in design uniformity and utility

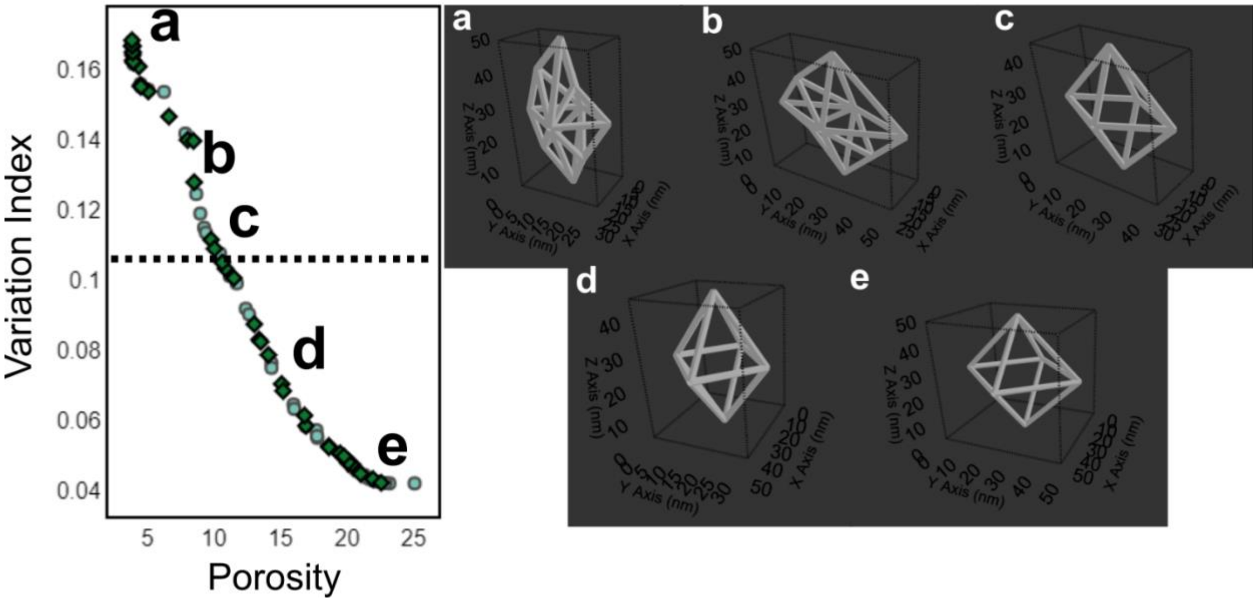

Figure S7: Sample generated solutions from the design uniformity versus utility multiobjective study (as described in the main article). Minimizing the edge length standard deviation tends to result in octahedral-like structures which become more regular as the variation index is decreased.

Supplementary Figure 8: AFM imaging and size validation of 2HB nanostructures

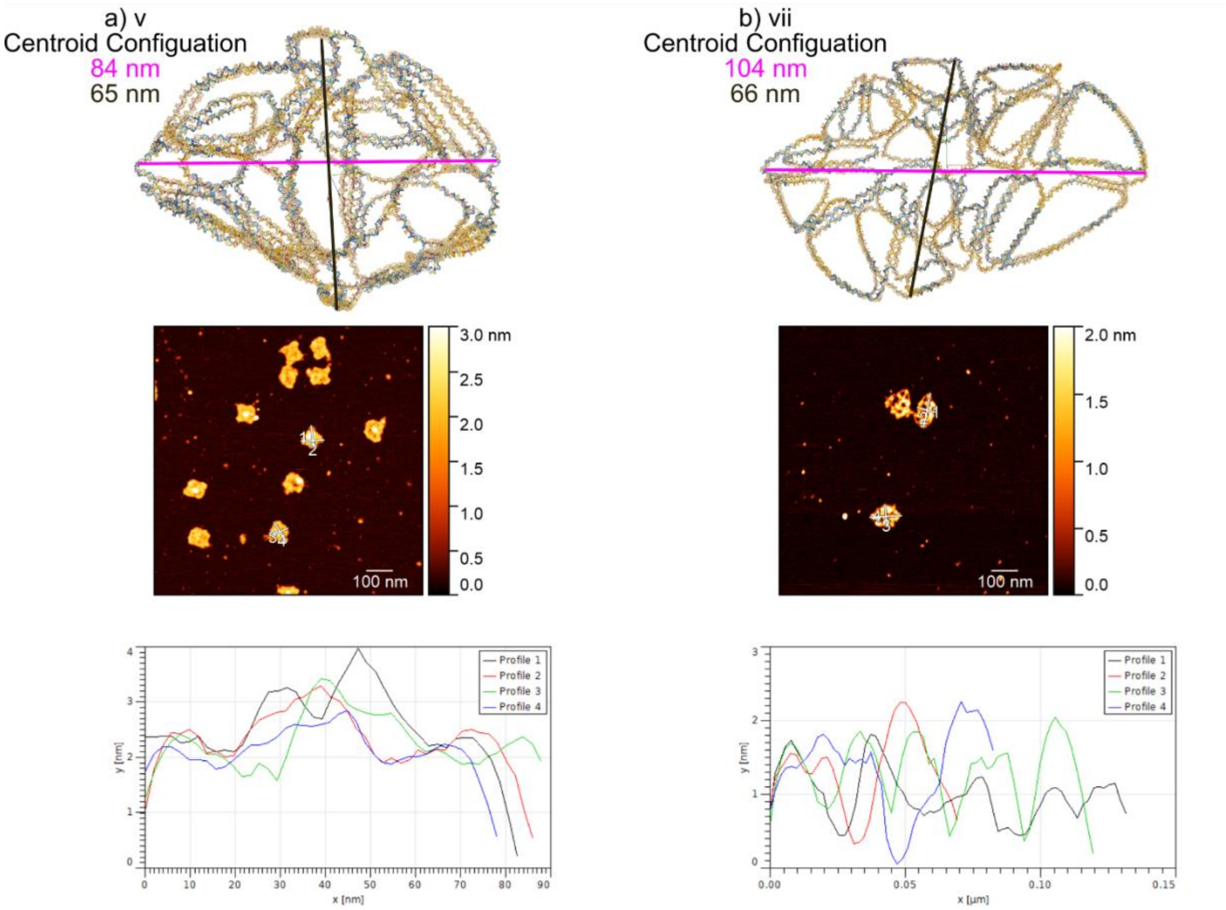

Figure S8: Measuring the observed nanoparticle sizes using Gwyddion (17). The centroid configuration found from the oxDNA simulation and visualized in oxView (13, 14) shows nanoparticle sizes of approximately a) 84 x 65 nm and b) 104 x 66 nm. While the observed nanoparticles vary in size slightly, the general structure appears similar and the deviation in size may be attributed to adherence to mica.

Supplementary Figure 9: AFM imaging and size validation of 6HB nanostructures

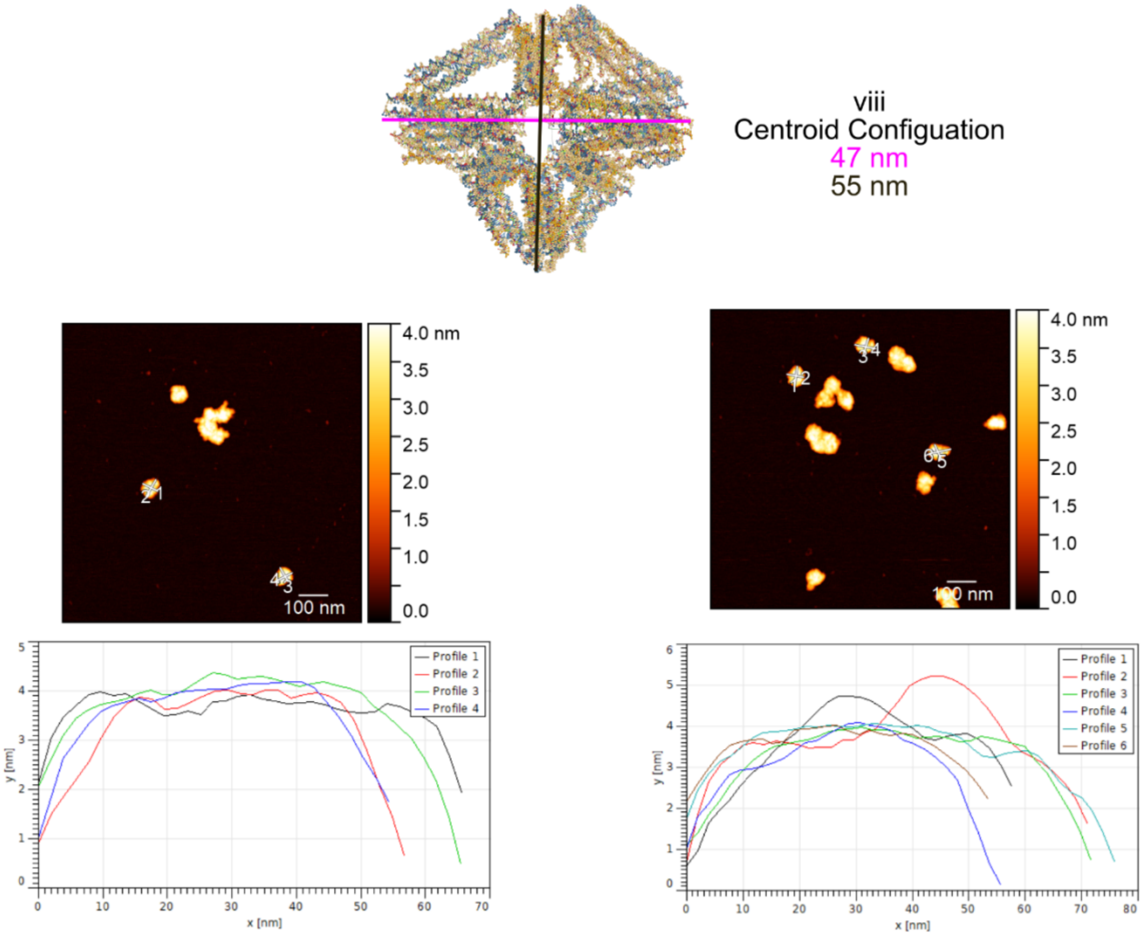

Figure S9: Measuring the observed 6HB nanoparticle sizes using Gwyddion (17). The centroid configuration found from the oxDNA simulation and visualized in oxView (13, 14) returns a nanoparticle size of approximately 47 x 55 nm. While the observed nanoparticles vary in size slightly, the general size of various particles are approximately the correct size as the simulated structure.

Supplementary Figure 10: Gel electrophoresis characterization

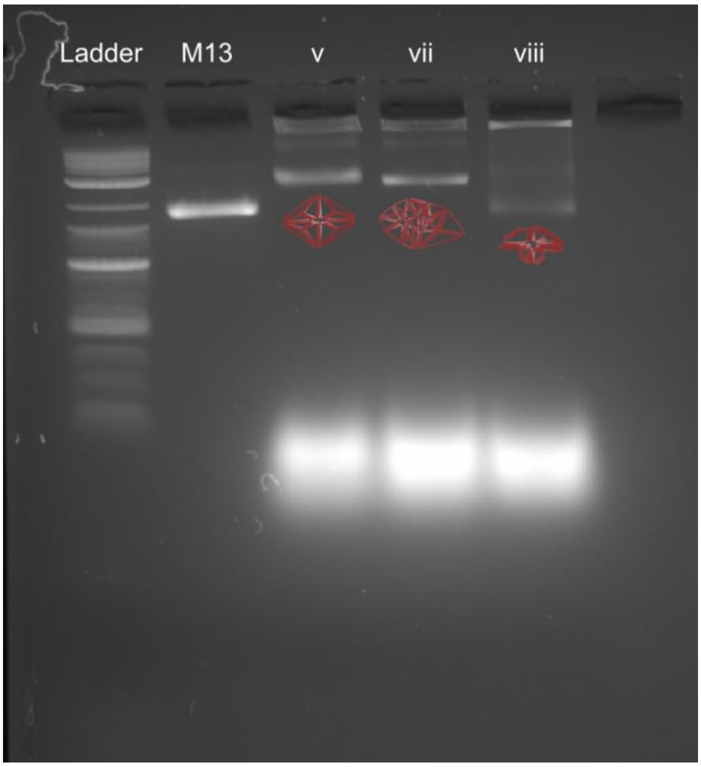

Figure S10: 2% Agarose gel electrophoresis of structures in 1x TBE and 12.5 mM $MgCl_2$ with 1x SYBR Safe DNA gel stain. From left to right: DNA ladder, M13, design v, vii, viii (identifiers found in Figure 6c of main article). 20uL of 20nM samples (including ladder and M13) were deposited into the wells. Gel was run for 2 hours at 70V.

Supplementary Table 1: DNA Scaffold and Staple Sequences for Design v

Below are the staple sequences for design vi from main article Figure 6. The M13mp18 scaffold was used.

Staple ID	# nts	Sequence
1	36	ATTAGCCATATGGTTTTTTTCAGCGCCAAAAAATTA
2	36	GCTATCTTGCAAGAAACAATGAAAGAGGGAGGGAAG
3	57	AATGAAAATAGCTTTTTTCTTTACAGAGAGTAACCCACAAGAATTTTTAAGCCCAAT
4	42	TATCAGAGAGAAATAACATAAAAACAGGGAAGTGAGCGCTAA
5	36	AATCAAGACTTGCGGGAGGTTTTGCACCCTGAACAA
6	42	GAACCTCCCGATTAGTTGCTATTTTGCACCCAGCGTTTTAGC
7	37	ATCGAGAATTTTTCAAGCCGTTTTTATTCAAGCAAAT
8	26	TTCATTATTTGTGAATTATCACCGTC
9	42	CAAACGTAGAATAGAAAGATTCATCAGTTGAGAGTATGTTAG
10	42	CCTTATTACGCATTTAGGAATACCACATTCAAGATTAAGACT
11	42	AGAACTGGCATCTAATGCAGATACATAACGCCAATACCCAAA
12	42	AATAATAACGGAAAAGGAATTACGAGGCATAGGAGGAAACGC
13	42	AATAGAAAATTAAGGCCGGAAACGTCACCAATTGTCACAATC
14	42	TAAGTTTATTTGAAACCATCGATAGCAGCACCCACCACGGAA
15	41	GTTTATCAACAATAGATTTTTCTGAACAAGAAAACGCACTC
16	42	AAACCAAGTACATAATATCCCATCCTAATTTAAACGGGTATT
17	36	AGCGTTTGATTTTCGGTCATAGCCCGGCTGTCTTTC
18	42	TTTTCATCGGCCCATCTTTTCATAATCAAAATCTGTAGCGCG
19	43	AAAGAAACGCAAAGAGTAATCAGTAGCGTTTTCAAGTTTGCCT
20	42	TACAGGAGTGTCCAGGCGGATAAGTGCCGTCGCTTTTGATGA
21	32	ATGGATTTTTCGCAGTCTCTGAATTTACCGTT
22	42	CTCATTAAAGCAAGACTTTTTCATGAGGAAGTCAAATAAATC
23	42	ATATTCACAAATTCCATTAAACGGGTAAAATAATTGGCCTTG
24	42	CAGGTCAGACGCGTAATGCCACTACGAAGGCAGAGGTTGAGG
25	25	AGTTAATGCCCCCTGCGGGGTCAGT
26	42	CGACAAAAGGTTTGAGAATCGCCATATTTAACTATAAAGTAC
27	36	CAGAACCGCGCCACCCTCAGAACCGCATTTTCGAGC
28	42	AGGTTTAGTACCCACCCTCAGAGCCACCACCCCGTACTCAGG
29	52	CCAGTAAGCGTCATACATGGAGAGGGTTGATATAAGTATAGTTTGAATAGGT
30	59	GGCTTAAAAAGTAATTCTTTTAGACGACGACAATAAACAACATGTTCAGCTCTGAGACT
31	50	GAGTTAATTTTTTGCCGCTTTTGCGGGATCGTCACCCTCAGCCCATGTTA
32	46	GTTGAAAATCTTGTATCGGTTTTTTTCAGCTTGCTTTCGATGCAGG
33	42	TCGCTGAGGCTGGTGAATTTCTTAAACAGCTTATATATTCGG
34	36	CAGAGGTGACGAACCACCAGCAGACAACAACCATCG
35	42	AAAAATACCGAAGGCGGTCAGTATTAACACCGCATCGCCATT
36	48	AAAACGCTCTCTGGCCAACAGATTTTTTTGATAGAACCCTTCTAGTCT
37	36	AAGAGGACGGGAACCGAACTGACCCACAGACAATAT
38	42	GAGATTTGTATTAGGCTGGCTGACCTTCATCAAAGTACAACG
39	42	AGCGCGAAACAAGAGTAATCTTGACAAGAACCGATTATACCA

40	42	AAAAGGGACATATGGAAATACCTACATTTTGAGACCAGTAAT
41	36	AGTAGTAAGCCCTGACGAGAAACATACATTGGCAGA
42	42	GAATAAGGCTTATTGGGCTTGAGATGGTTTAACTCATTCAGT
43	42	CCAGAGGGGGTCCGAAAGACTTCAAATATCGCAGAAGTTTTG
44	42	GCTTTTGCAAAGTTTTAATTCGAGCTTCAAAGATAGCGAGAG
45	25	TGAATCCCCCTCAAATAGCGTCCAA
46	40	GCTTTCGACAGGAACGTTTTTTTGTACGCCAGAGTAATAA
47	42	TTCTTTGATTAATCCTGAGAAGTGTTTTTATAGTAGCAATAC
48	36	CATTTTTGTAATTGCTCCTTTTGATCTGTCCATCAC
49	42	GAGAGTACCTTCGGATGGCTTAGAGCTTAATTGTCAGGATTA
50	36	TATCCAGATCAAACTATCGGCCTTTGACTATTATAG
51	36	CCTTTAATCCAAAAAAAAGGCTCCGGAAGGTTATCT
52	42	AACAACTAAAGACGATCTAAAGTTTTGTCGTCAATAGAAAGG
53	42	AGCGGAGTGAGTTTCCAGACGTTAGTAAATGACAACAGTTTC
54	25	AGGCGAATTATTCATTTGATTGCTT
55	25	TAGACTTTACAAACAACGTCAATAG
56	42	CCTCAAATATCCGTTATTAATTTTAAAAGTTTCCTTGCTGAA
57	42	CTAAAGCATCAGAGTAACATTATCATTTTGCGAATGAAAAAT
58	45	AATAGCCCGAGAAAGGAAGGGTTTTTTGAAAGCGAAAGGATATCA
59	42	TAGAACCTACCAGCGGGCGCTAGGGCGCTGGCATGGAAGGGT
60	42	ACTTCTGAATAAAGTGTAGCGGTCACGCTGCGTTTGGATTAT
61	42	AATCCTGATTGCGTAACCACCACACCCGCCGCTCATCAATAT
62	42	ATGATGGCAATGCTTAATGCGCCGCTACAGGGTGATTATCAG
63	36	AGCAGAACAAAGAAACTTTTTCCAGAAGGAGCGGAA
64	42	TGTAAAACGACAATAAGAAACGATTTTTTGTTGTCACGACGT
65	42	GCGAGCTGAAATGCGAACGAGTAGATTTAGTTCATTTGGGGC
66	36	GAAAATCCCCACGCTGGTTTGCCCGTCAATAACCTG
67	25	GAGCTCGAATTCGTAATGCCTGCAG
68	42	TGCCAGTTACAATTGTTATCCGCTCACAATTCGAGCCTAATT
69	42	GCGTCTTTCCACACACAACATACGAGCCGGAAACGCTAACGA
70	42	GAATCGGCCAAAACGCGAGAAAACTTTTTCAACTGCATTAAT
71	42	TGTCGTGCCAGATATATTTTAGTTAATTTCATTCGGGAAACC
72	42	CCGCTTTCCAGCTTCTGACCTAAATTTAATGGCGCTCACTGC
73	42	TAATTGCGTTGTTTGAAATACCGACCGTGTGATAACTCACAT
74	41	TTTTATCCTTTTACCAGCATAAAGTTTTAGCCTGGGGTGCC
75	36	TGAGAGATCAATATATTTTTGAGTGAATAATTTTCC
76	25	GTACAAACTACAACGCGGAACCCAT
77	26	CTTAGAATTTTTGAAAACATAGCGAT
78	42	ACAGTACATAAACTACCTTTTTAACCTCCGGCTTTAATGGAA
79	42	GAATTACCTTTTTAGGTTGGGTTATATAACTAAATTTCATTT
80	44	ATAATGCTTTTTGTAGCTCAACATGTTTTAAATATGGTCTATCA
81	36	GTAAAGCACAAGTTTTTTGGGGTCACAGGAGGCCGA
82	42	GAGTCCACTATCGATTTAGAGCTTGACGGGGATTTGGAACAA
83	42	TGTTGTTCCAGAAGCCGGCGAACGTGGCGAGATAGGGTTGAG
84	48	GTAAATATTGACGGGACAAAAGGGCGACATTCAACCGATTTAGCAATA
85	48	AGTCAGAGGGTAATCGCATTAGACGGGAGAATTAACTGAAAAGCCTTA

86	44	CAGATATAGATTTTATCCGGTATTCTAAGAACGCGAGGCTACAA
87	39	ACCGACTTGAGCCATTTACCGCGCCCAATAGTTCATCGT
88	49	AGGAATCATTGGGAATTAGAGCCAGCAAAATCACCAGTAGTCCATTACC
89	48	CTTATCATTCCAAGCGAGCATGTAGAAACCAATCAATAATCCCTTATT
90	32	TGAAACAGAAGGATTAGGATTATTGGGTTTTG
91	47	TTAGCGTCAGACACCGGAACCAGAGCCACCACCGTTTTTTGAACCGC
92	59	GCCTTGAGTAACAGTGCCAGAACCGCCACCCTCTCAGAGCCGCCACCCTCCGTATAAAC
93	33	GAAAAAGCCACCAGTATAAAGCCATAACAGTAG
94	48	CAGTAATAAGAGAAAACGCCAACATGTAATTTAGGCAGAGGCCACCCT
95	48	CTCAGTAACTGGTAATATTTTTTAACCTATTTCGGAATTTTATTATTC
96	40	CCTCAAGATGAAAGTATTAAGAGGAATGCAGAACGCGCCT
97	46	CTTAGCCGAAATCCGCGACCTGCTAGCGAAAGACAGCATCGGAACG
98	48	CCCACGCATAACCGGATACCGATAGTTGCGCCGACAATGAAGATAAAA
99	48	TTTTGAATGGCTATTGACCTGAAAGCGTAAGAATACGTGGAACTTTGA
100	40	AGGCGCACATCGCCTGATTTTAATTGTGTCGGAACGAGGC
101	56	CATCACTTGTTTTGAGTAGAAGAACACAATATTACCGCCAGTTTTTTGCAACAGGA
102	48	TTCACCAGTCACACCGCTCAATCGTCTGAAATGGATTATTCCAGAACG
103	58	TTGACCCCCAGCGGATATTTTTCCCAAATCAACGTAACAAAGCTGTTTCAACTTTAAT
104	47	ACAGTTCAGTTAACGAGAATGACCGATTGCATCAAAATTTATTAAGA
105	59	TACTGCGGAATCGTCATTTGGGAAGAAAAATCTTATACCAGTCAGGACGAAATATTCAT
106	40	GGAAGCAATAGTAAAATTTTGTTTAGACTGGATGCTTTAA
107	48	GCAAATTAACCGTTATCAGTGAGGCCACCGAGTAAAAGAGTAAGAGGT
108	51	AAAAACCAAACGAACCAGATTTTTTCGGAAGCAAACTCCAACAGGCTGAAT
109	48	TCAGAAGCAAAGCGATAAATCAAAAATCAGGTCTTTACCCGCTGGTAA
110	47	CTCGTATTATTTCCTTTGCCCGAAAAACCCTCAATCATTTATCTGGT
111	48	AAAATATCTTTAGGGCAAATCAACAGTTGAAAGGAATTGAAAAAGGAG
112	59	TGAATACCAAGTTACAATTTGCTAAACAACTTTATTTTCTGTATGGGATAATCGCGCAG
113	59	ATAATACATTTGAGGATGATTTTCAGGTTTAACATAAAGAAATTGCGTATTAGAAGTAT
114	41	CAGTTGAGCACTAACATTTTAATAGATTAGAGCTTCGACAA
115	32	GTGTGAAAAATAAACAGCCATATTATTTATCC
116	59	GGCATTTTTCTACTAATAGTAGTAGCATTGTAACGCCAGGGTTTTCCCATAACGTCAAA
117	48	TTTAGCTATATTTTTGACCATTAGATACATTTCGCAAATGCAGCAGGC
118	59	GTCGACTCTAGAGGATCTTTTTCTTTTCACCAGTTGGGCGCCAGGGTGGCCCGGGTACC
119	48	CAATCCAGGCCAGTGCCTTTCTTGCATCATGGTCATATTTTGTTTCCT
120	50	AATTAATTACATTTAACTATGTAAATGCTGATGCAAATTTTTATCGCAAG
121	59	GTACCGTAACACTGAGTCGTCGCTATTAATTAACCTTGCTTCTGTAAATTTCGTCACCA
122	57	TAATGAGTGAGCTAAATAAGGCGTTAAATAAGAATAAACACCGTTTTATAATTACTA
123	39	AGCTTAGATTAAGACCGTTATACAAATTCTTTGTTTAGT
124	49	ATCATATGGCTGAGAAGAGTCAATAGTGAATTTATCAAATTTATAGGTC
125	46	GGGCGATGCAAAGGGCGAAAAACCCAACTAAAGTACGGTGTCTGGA
126	53	TTATCATCATATTCCCGCGTACTATGGTTGCTTTGACGAGTTTTTTATAACGT
127	48	TTAAAGGGATTTTACTCGTTAGAATCAGAGCGGGAGCTAAGAGGTGCC
128	42	AGGGTAGCAACGGCTACAGAGGCTTTTTTTGAGGACTACAGA
129	45	ACAGACAGCCCTCTTTAGCGTAGAATTGCTTTATAATTTTTTCAC
130	42	GTATCACTCATTTTCAGGGATATTCCAATACTGTAGCATTCC
131	42	ACGGATTCGCCTCAATTACCTGAGCAAAAGAAAGAAACAATA

132	41	AAATTATTTTTTTTCACGTAAAACAGAAGTCAGATGAATAT
133	42	CAGCAAGCGGTTGTTTGATGGTGGTTCCGAAAGAGAGAGTTG
134	51	ACAAAGCGCGCGGGGAGATTTTTTTGCGGTTTGCGTATGAGACGGGCAACA
135	47	ACAGTAACAGTACCTATCGGGGATGATTTTAAACATCAAGAAAACAA
136	48	GCTGATTGCCCTTCATTTTTGGCCCTTCGGCAATTTATAAATCAAAAG
137	59	TTAATGCTTAACTGATAGCCCTAAAACCTGCAACAGTGCCACGCTGATTTTTTGCCAGC
138	54	GAACTAACGGAACAATTTTACAGGAATACATACTTTTTAAGGTGGCAACATATA
139	42	AGCATTGACAGCCAACCTAAAACGAAAGAGGCAGCCGCCGCC
140	20	CTCCCAGAGCCACCACCCTC
141	53	AGAGCCGCCACCAGAATTTTTACCACCAGAAAAGAATTTTAAAACACTCATCT
142	46	CATTGTGAATTACCTTTTTAAGAACTGGCTCATACGTTAATAAAAC
143	42	AGAAGGAAACCTAAGAGCAACACTATCATAACCAAAGTTACC
144	21	AATAAGAACCGAAGCCCTTTT
145	45	TAAGAAAAGTAAGCATTTGCCGAACCTCGTTTTCCAGACGACGAT
146	41	AGTTTCATTCCATATAACAGTTGTTTTTTCCCAATTCAGGT
147	38	GCAGACTTTCAATCATAAAGATGAACGGTTTACAGACC
148	42	GAGCCCCTAAAGAACGTTTTTACTCCAACGTGCCCACTACGT
149	36	GAACTTTCACCCAAATCTAAATCGGATTCCTAAAGG

Supplementary Table 2: DNA Scaffold and Staple Sequences for Design vii

Below are the staple sequences for design vii from main article Figure 6. The M13mp18 scaffold was used.

Staple ID	nts	Sequence
1	44	TCGGGGCAGAGGCATTTTCGATGTAATAAGAGAATATAAGACGA
2	40	TCGCCGCGAGAAAACTTTTTTAATATATTTTAGTTGAAAT
3	53	TTCCCTTAGAATCTTTTGAAAACATAGCGATAGCTTAGATTAAGACTAAATTT
4	42	CATGTAATTTACTGTCTTTCCTTATCATTCCAACAACGCCAA
5	42	TACCAGTATAAAGTGAGAATAGAAAGGAACAATACAAATTCT
6	42	CATATGCGTTACTAAAGGAATTGCGAATAATATGTTTAGTAT
7	53	TGCCGGGCCTTCCTGTTTTCCAGCTTTCATCAACATTAATGCAAAAGAAGTTT
8	42	AATTCGCGTCTGAAACCAGGCAAAGCGCCATTCATCAAAAAT
9	36	GTCATAAAGATAGCGTCCAATACTCGTTAATATTTT
10	59	ATATAACAGTTGATTCACAAAATTAATTACATTTTCAATTTCATTTGAATATATATGTG
11	27	AACAGTTTTTTTACATCGGGAGAAACA
12	53	TTAATGGAATTGCTTTGAATACCAAGTTACAAAATCGCGTTGCGAATTATTCA
13	59	CGCGAGCTGAAATTTGTGGCATCAATTCTACCTGAGCAAAAGAAGATGTGAAACAAACA
14	36	GCATGTCACGATGAACGGTAATCGAGCTCAACATGT
15	25	CTGTTTAGCTATATTTTTGACCATT
16	36	CGCACTCCCGACAGTATCGGCCTCCCGGGTACCGAG
17	54	AGTAACAACCCTTGGATTCTCCGGGGCGCATCGTAACCGTTTATCTGCCAGTTT
18	36	TTGACGCTAACGCTCATGGAAATAATGGGATAGGTC
19	46	TGAGAAGTGTTTTTTTTTTATAATCAGTGAGGCCACCGAAGAACAA
20	42	TGGTAATATCCGTAAAAGAGTCTGTCCATCACTCGGCCTTGC
21	42	AACTCAAACTAGCAAATTAACCGTTGTAGCAATGAGTAGAAG
22	25	TAATGAGTGAGCTAACCGAGCCGGA
23	42	CACACAACATATCACATTAATTGCGTTGCGCTCTCACAATTC
24	40	CAAGCTTGCATCATGGTCATAGCTTTTTTCCTGTGTGAAA
25	50	TCAATAGATAATTTTTTTCATTTGAGGATTTAGAAGTATTGCCAGAATCC
26	42	AGGAACGGTACAGACTTTACAAACAATTCGACGATTTTAGAC
27	42	CCGATTAAAGGAACTCGTATTAAATCCTTTGCAAACAGGAGG
28	25	ACGTGCTTTTTTTTCGTTAGAATCA
29	52	GGCGAACGTTTTCGAGAAAGGAAGCTGGCATTTTGTAGCGGTCACACGTATA
30	42	CTTTGACGAGCGCTGCGCGTAACCACCACACCACTATGGTTG
31	25	CGTATTGGGCGCCAGGTAATGAATC
32	54	TAAATCAAAAGAATAGCCAACGTGGACTCCATTTTTGTCAAAGGGCGAATTCAA
33	42	TCAATATGATAAAACCGTCTATCAGGGCGATGCAAATCACCA
34	42	CCGGAGACAGTGCCCACTACGTGAACCATCACGTGAGAAAGG
35	42	GATTCAAAAGGCCAAATCAAGTTTTTTGGGGTTGTAGGTAAA
36	42	CCTGAGTAATGCGAGGTGCCGTAAAGCACTAAAAATGCAATG
37	25	CCCTGACTATTATAGTAACGAGAAT
38	56	AATAGTGAATTTATCTTTTAAAATCATAGGTCTGAGAGACTACCTTTTTAAGCGAA
39	58	ATTTCAACTCATTGTGAATTACCAAGAGGAAGCCCGAAAGACTTAAATATCGCGTTTT
40	54	TGAATCCCCTTAATGCTTTAAACAGTTCAGAACAGAAGCAAAGTTTTGCATCAA

41	42	AAGACAAAGAACCACGCATAACCGATATATTCATCCAATCGC
42	51	AATTCGAGCTTCAAACCTCCGGCTTAGGTTGGGTTTTTTAACTATATGTAA
43	56	ATGCTGATGCAAGGTCGCTGAGGCTTGCAGGGAGTTTTTAAAGGCCGCTTGGTTTA
44	53	AAAGATTTTATGCGATTTTAAGAACTGGCTCTTTTTATTATACCAGTCAGGAC
45	43	GCTACAGATTTTTTTGAGGACTAAAGACTTTTTCATGATGCCA
46	26	AGCCACCACCTTATTTTCAGGGATAG
47	43	ATAAACATTTTTAATGCCCCCTGCCTATTACCGCCACCCTCAG
48	59	CAAAATCACTCTGAATTTACCGTTCCAGTAAGTTTACATGGCTTTTGATGATACCCCGT
49	36	GACCCCCAGAATACACTAAAACACCGGGGTCAGTGC
50	35	CATTCCACCAGTACAAACTACAACGGAGCCTTTAA
51	42	TATCACCGTACGTCGTCTTTCCAGACGTTAGTGGAATAGGTG
52	49	CAGAACCACCATTTTTTCCAGAGCCGCCGCCAGCATTGACAGTACCAGG
53	42	GGGGTTTTGCTCAGGAGGTTGAGGCAGGTCAGTAGGATTAGC
54	42	AAGAGAAGGATACGATTGGCCTTGATATTCACGAGACTCCTC
55	36	CATCGGCATAGCGTCAGACTGTAGGACCTGCTCCAT
56	54	AGAGAATTTTAAAAACAGGGAAGTAGCAGCACCGTTTAGTAGCGACAGAATCAA
57	42	GAAACCATCGACGCATTAGACGGGAGAATTAACGTCACCAAT
58	42	AAGGCCGGAAACTGAACACCCTGAACAAAGTCTACCATTAGC
59	25	CTGACCTTCATCAAGACAGATGAAC
60	59	TAAATCCTTTTTAAAGCCAGAATGGAAAGCGCAGTCCGGAACCAGAGCCACTTCATAAT
61	42	CCACCACCCTCAAACGATTTTTTGTTTAACGTACCCTCAGAG
62	42	TCAGAACCGCCCAAAAATGAAAATAGCAGCCTGCCGCCACCC
63	36	CTAGGGCGGGAAGAAAGCGAAAGGTTTGGAACAAGA
64	54	GAGCGGGAGCTCCGAACGTTATTAATTTTTTTTTAAGTTTGAGTAAGGAAAGCC
65	42	GAGCTTGACGGCATTATCATTTTGCGGAACAACCCCGATTTA
66	59	CCGCCTGCAACAGTGCCATATCTGGTCAGTTTTTTATCAACAGTTGAAAATTAGAGCCG
67	36	CCTGATTGATGATGGCAATTCATCTCTTTAGGAGCA
68	42	ACCCTCAATCAACGCTGAGAGCCAGCAGCAAACAAATATCAA
69	25	CCTCCCGACTTGCGGGATCCGGTAT
70	25	GAACCCTTCTGACCTGCCAGTAATA
71	42	AACGCCAGGGTCCTGGCCCTGAGAGAGTTGCATAAGTTGGGT
72	42	TGCAAGGCGATGCAAGCGGTCCACGCTGGTTTGGGGATGTGC
73	42	TTCCAGAGCCTTAGTTGCTATTTTGCACCCAGACGAGCGTCT
74	25	GCAATAGCTATCTTACTTAAGCCCA
75	44	AAAACGAACTTTTTTTAACGGAACAACATTATTACAGGCCACAT
76	41	AGAATACGTTTTCAGACAATATTTTTGAATAAATACATACA
77	40	AGTGAATAACCTTTTTCTGTAAATCGTCGCCAGAACGCGC
78	57	ACCGACCTTTTTTTGATAAATAAGGCGTTAAATTAAAGTAATTCTGTCCAAGTACCG
79	52	CTGTTTATCAACTGATAAGTCCTGAACAAGATAGAAACCAATTTTTCAATAA
80	49	ACAAAAGGAAGAATAAACACCGGAATCTTACTAGAAAAAGCCATTTTTT
81	50	GTCAGATGAATATCTAATTTACGAGCATGAAAATAATATCCCATCACAGT
82	56	CCCTCGTTTACCATTTTACGATAAAAACCAAAATAGCGAGAGGCTTTATGTGAGCG
83	37	GAGGGGACGAAGCCAGCTTTCCGGCACTTTTTTCTGG
84	48	GTTAAAATTCGCATGTATAAGCAAATATTTAAATTGTAAAGCGGAATC
85	40	ATAACGGATTCGCCTGAACAGTACATAAATCATACCTTTT
86	48	TTTAAATATGCAACAGCTTAATTGCTGAATATAATGCTGTTAAAACTA

87	59	AGATACATTTCGCAAATTTTTTGAGAGATCTACCCGGAGAGGGTAGCTAGGTCAATAAC
88	46	TCAAGAAACCAATTCTGCTTTTCGAGTAGATTTAGTTCATTTGGGG
89	48	CTCGAATTCGTAATGCCTGCAGGTCGACTCTAGAGGATCCAGGAAGAT
90	48	ACGTTGGTGTAGATTGGGAACAAACGGCGGATTGACCGTACCTACATT
91	47	TATTACCTTTTTTGCCAGCCATTGCAACAGGAAACAATCGTCTGAAA
92	59	AGCATAAAGTGTAAAGCAATAACATCACTTGCCTACTTCTTTGATTAGTCTGGGGTGCC
93	59	GGCCAACGCGCGGGGAGCCGCTACAGGGCGCGTCGCCGCGCTTAATGCGAGGCGGTTTG
94	59	GACCATAAATCAAAAATAGAGTACCTTTAATTGAACAGGTCAGGATTAGCAGGTCTTTA
95	54	TTGTATCGGTTTCTTGCTTTCGAGGTGAATTACAATGACAATTTTTTTCAACCA
96	53	CAAGCCCAATAGGCGGGTAAAATACGTAAGGAAGTTTCCATTAAAAACCCATG
97	48	TCTAAAGTTTTTCAGGAGGTTTAGTACCGTTTTCCTCAGAACCGCCAC
98	48	CTTGAGTAACAGTGAGGAGTGTACTGGTAATAAGTTTTAATCATCTTT
99	50	CTACGAATTTACCAACCTAAAACGAAAGAGGCAAAAGCGATTATACCAAG
100	36	CACGTTGAACAAAAAAAAGGCTCCAAAAGCCTGTAG
101	52	CGGATAAGTGCTTTTGAGAGGGTTGATATAAGTATAGCCCAAATGAATTTTC
102	48	GTTACTTAGCCGGAGCCTGATAAATTGTGTCGAAATCCGCCGCGTTTT
103	25	GTTTGCCTTTTTTCGGTCATAGCCC
104	43	GGCATGATTAAGACTCCAACCGATTGAGGGAGGTTTTGGTAAA
105	59	GGTGTACAGACCAGGCGTAAAGGTGAATTATCAGACGGAAATTATTCATCATAGGCTGG
106	45	CGGTCAATCATAATCGAACTGACCAACTTTGAAAGAGGAGTAATC
107	54	CCTTATTAGCGTTTTTTTGCCATCTTCACCGGAATTTCCTCCCTCAGATTACAG
108	48	GTCCACTATTAAAGCGAGATAGGGTTGAGTGTTGTTCCAGAGCGGGCG
109	48	CTAACAACTAATAGGGAATTGAGGAAGGTTATCTAAAATAAATATAAT
110	59	TCTAAGAACGCGAGGCGTCACCTTGCTGAACCTTGAAAAATCTAAAGCATTTTAGCGAA
111	59	AAAGGGACATTCTGGCCAGGCATAGTAAGAGCAGCCAAAAGGAATTACGAACAGAGATA
112	59	ATAATAAGAGCAAGAAAAATCTTACCAACGCTACTACAATTTTATCCTGCAATGAAATA
113	55	TAAAGGTGGCAACTTTTTATAAAAGAAACGCTTGAGATTTAGGAATATAGAAAGA
114	35	TTGACAATTTTTTGAACCGGACAATAGAAAATTCA
115	58	TCTGAATAATGGAATTTAGAACCTACCATAGAAATTGCGTTTTATTTTCAGGTTTAAC
116	54	AACCAAGTACCGCACTTTTTATCGAGAACAAGCAAGCCGTTTTTATCGTAAAAC
117	52	TTTCAATTACTAATAGTAGTAGCATTATAAAAATTTTTAGATTTTTTCATAT
118	38	ATTTTATCGGATTTTTCTAAAGGGAGCAGAAACCACCA
119	52	GAAGGAGCGGAATTTTTTCATCATATTCCTGATTATCAGTTTGGATTATACT
120	47	TTGTTATCCGCACTGCCCGCTTTCCAGTCGGGAAACTTTTGTGCCAG
121	55	TTGCCCTTCACCGTTTCCCAGTCACGACGTTGTAAATTTTTTCGACGGCCAGTGC
122	44	CTGCATGTGGTTTTTCTTTTCACCAGTGATTGGGCAACAGCTGA
123	29	CAGAGGTTTTTTGGCGGTCAGTATTAACA
124	36	ATGCGCGAACTGATAGTTTTTAACATCGCCATAAAA
125	42	TGGATTATTTACATTTTATTCACCAGTCACACGAAAAGCGTA
126	54	GGCGACATTCTTATTACGCATTTTGTTAGCAAACGTAGAGGCTATTAGTCTTTA
127	52	CCTCAGATCGGAACCTATTATTCTGAAACATTGTATTAAGAGGCTAAACAAA
128	40	CGACAATATTTAACATGTTCAGCTAATGTATTAATTAATT
129	28	ACGGTGTTTTTTCTGGAAGTTTCATTCC
130	34	GAGGTCATTTTTGCGTTTTTTGGCTTAGTAAAGT
131	39	CCAGACCTTTTTTGGAAGCAAACTCCCTCCTTTTGATAA
132	38	AATGGTTTAATTTCATCTTCTGACCGCTGAGAAGAGTC

133	39	TGCCAGAGGGGGTAATTAAAATGTTTAGACTGTATTCAT
134	36	TCAACTATTTTTCAGATACATAACACACTATCATAA
135	54	CAAATCAACGTATTTAGCTGCTCATTCAGTGAATAAGGAAAAATCTACGTTAAT
136	42	AATAGGAACGCCGCCATTCAGGCTGCGCAACTTTTTTTAACC
137	36	CCGTTCTTTTTTGATAAATTAATGAAAGGCTATCAG
138	42	GCTGGCGAAAGGCCCCAGCAGGCGAAAATCCTTATTACGCCA
139	50	GAAGATTTAAATTTTTTTTATCAGCTCAGTTGGGAAGGGCGATCGGGCCT
140	29	GTTGATAATCAGAAAAGTTTCAAAAACAG
141	52	GTCATTGCCTGAGTTTTTCTGGAGCAAACAAGAGAATATCATATGTACCCCG
142	45	CTTCGCGTTTGATGGTGGTTTTTTTTAAATCGGCAAAATCCCTTA
143	52	TGTATGGGATTTTGTTTACAACTTTCAACAGTTTCAGCGGAGCCAACGCTCA
144	50	ACAGTAGGGCTTTTTTAATTGAGAATCGCCATATTTAAGAACGGGTATTA
145	52	TACAAAATAAACATTTTATTATTTATCCCAATCCAAATAAGAGAGCCGCCAC
146	33	GAATCATTACCGCTTTTCAATAGCAAGCAAATC
147	35	AGAAATAAATCAAAATTATTTGCATTTCATCGTAG
148	52	AGATATAGAAGGCTTAGGTTTTGAAGCCTTTAAATCAAGATAATTTGCCAGT
149	45	GTTGGGAAGCTTGCCCTGACGAGAAACACCTTTCGAGTAGTAAAT
150	52	TGGGCTTGAGATTTGCGGGATCGTCACCCTCAGCAGCGATTTCAGCATCGGA
151	46	CGCGAAACAAAGTACTTTTGAGATTTGTATCATCACGAGGCGCAGA
152	52	TTCATCAGAAAGACACTTGAATAAGTTTATTTTGTCACAATTATTCATTACC
153	47	ACGAGGGTAACCGATAGTTGCGCCGTCTTAAACAGCTTGATGCAACG
154	47	TATTCCGTCACCGACTTGAGCCATTTGGGAATTTCAGCAAAATCACC
155	46	AGTAGCACCATAGAGGGTAATTGAGCGCTATTTAGAGAGATAACCC
156	55	ACAAGAATTGAGCGAAGCCCTTTTTAAGAAAAGTAAGCAGATAGCTTTAAGTTAC
157	35	GAAACGCAATAATTTTTGGAATACCCAAAAGAACT
158	47	CAGAAGGAAACAACCACCAGCAGAAGATTAAAAATACCGAACGCGAG
159	48	TACCGTAACATTTTGTTTCGTCACAGACAGCCCTCATAGTCGTAACGA
160	29	TATGGTTTACCAGCGTTTAAAGACAAAAG

Supplementary Table 3: DNA Scaffold and Staple Sequences for Design viii

Below are the staple sequences for design viii from main article Figure 6. The M13mp18 scaffold was used.

Staple ID	#nts	Sequence
1	41	GTAGCATTGAGATCTAAATTCTATTTCACGTTATTTTCATT
2	46	AAAAGGAATTACGATTACAGGTAGAATTCAACTAATGCAGTTAACG
3	41	CTTACCAACGCTAACGCAATTTTTAGGAATCATTAAATCAA
4	41	ATCAATAATCGGCTGTGAGCATGATCAGAGAGCCTGAACAA
5	46	AAATGCTTTAAACATAGTAAAATGTTTCATAAATATTCATCGTGCC
6	40	AGAATAACATAACATAGTAAGAGCTTTTTTTAACACTATC
7	52	TTTTAATAATAACCCAGATGAATAGGAAGATTGTATTTTTTTTAAGCAAATA
8	35	TCAAACTATGCAGATTCACTTTTTTTCAGTCACAC
9	40	ACAGGAAAAACGAAATTTTTGTTATTTTTTTAATCAGCTC
10	35	CAGGGCGATCACCACACCCTTTTTTTGCCGCGCTT
11	35	ATTTGGGAAAGCCCCCTTATTTTTTTTTAGCGTTT
12	41	GCACCGTAATCAGTAGGAAACCATAAGAGGCTACCATTACC
13	52	AGCGTAAAGTAAATTAGGTGTATACCTCCGGCTTAGTTTTTTTGTTGGGTTA
14	40	ACCACCAGAGCCGCCCTTTTTAAGTTTTTTTAAAAGTAAG
15	42	GAACCAGCGCGTTTCCAGTTTGAGGGGAATTGACCGAGCGAG
16	58	TTTAGCTATGAATTCTGCGAACGAGTATAACATATCAGGTCATTGCAAGAGCTGAAAA
17	39	CCTCAGAAGAGGGTGCTAAACAACTTTCCAGCCTGTAGC
18	51	CGTCTTTCCAGACGTTCGATCTATGAATTTATCCGTAACACTGACATAGTT
19	39	AAACAATAAATTGCATTAATTAATTTTCAGAATTACATT
20	51	TCAATATATGTGAGTGGGAAACATCATATGTAAAAGAAGATGATTTACCTT
21	58	TTTGAAGCCTTAAGGCTTATCCGGTAGCAAGCTTCCAGAGCCTAATTAGCTATTTTGC
22	58	TAGATAAGTATTGTCCAGACGACGACAAAAGGTATCATTCCAAGAAAAATATCCCATC
23	39	GGATGTGGCTGCGCCCTGGGGTGCCTAACTAGCTCGAAT
24	51	CACAATTCCACACAACGAAATTGAAATCCTGTATGCCTGCAGGTGCTGTTT
25	39	TTTTGATAGCGCAGTACCTTATGCGATTTTTTGCCCTGA
26	39	AACCGATGTGAATTAAACACTCATCTTTAGTAAAGACTT
27	51	ACGAAGGCACCAACCTAATACGTATAAATCAAGGAACGAGGGTACATTAAA
28	39	TCCAACGGTTGTTCAGCGAAAGGAGCGGCTTCGGAACCC
29	39	TAAAACATAGTTAAATAATACATTTGAGGAATCTGGTCA
30	51	ATCTTTAGGAGCACTAGAAGGTTGTCTTTAATCACCTTGCTGAATGAAAGG
31	58	ACCAGTAGCGACATTAAAGGTGAATATATTGAATCAAGTTTGCCTTTTGGCCGGAAAC
32	42	CAAATATACCGGAACTTAGAGCTTAATTTAAATGTGTAATGG
33	32	TAAAGTATTTTTTTCGGTGTCTGTCTGGAGCA
34	32	TGCTCAGTTTTTTTTACCAGGCGTCAGCGGAG
35	39	ACCCATGTACACCGTACTCAGGAGGCCCGGAAGAATTTT
36	44	AGAAACCACTCAAAATTATTTTTTTTTGCACGTAATCCTTGAA
37	39	ACCTGAGCACCACAGTAACAGTACCTTAACGTTTGCTTC
38	44	AGTTTTAACAATCTACGTTTTTTTTTAATAAAACGGGGAGAATT
39	51	AACTGAACACCCTGAAATTAGACAACTAACGGAACAACATTAGGAAACAGG
40	44	CAATACTGCTTGCCCTTCATTTTTTTCCGCCTGGCTTACAAAAT
41	44	AGCCTGTTTTCGAGCCAGTTTTTTTTAATAAGAGATAAACCAAG

42	49	AGATGAACGGTGCGAGAGGCTTTTTTTTTTTGCAAAAGAAGTTTTTCTT
43	51	TTCACCAGTGAGACGGCAGGGTGGTTTTGCCAGAGGGGGTAAGTTGCGTAT
44	44	ATTGCTCCTCGGAAACCAGTTTTTTTGCAAAGCGCGCTAACTCA
45	39	CAAGCTTGCTTTTCGCTATTACGCCGCGATCGCCGGAAG
46	44	GGGAGTTAAGATTTGTATCTTTTTTTATCGCCTGAGGCTGACCT
47	51	TCATCAAGAGTAATCTATAGGCTTAAATTGTGTCGAAATCCGAGTACAGAC
48	44	CCACCCTCAAACAAATAAATTTTTTTTCCTCATTACTGGCTCAT
49	45	CGAGAAACACTGTGAATTCTCTGAATTTACCGTTCCTATCAACTT
50	32	ATTGTATTTTTTTTCGGTTTATCAGCGATTAT
51	39	GACAGCATCAAACAATGACAACAACATACCGAAAGAGGC
52	44	AGAATCAGAGCAAATTAACTTTTTTTCGTTGTAGCTGACGCTCA
53	51	ATCGTCTGAAATGGATTACATTTAATACTTCTTTGATTAGTATTCTCATGG
54	44	ACCGCCTGCTTAAATCCTTTTTTTTTTGCCCGAACTACTTCTGA
55	51	ATAATGGAAGGGTTAGTGGATTAGTTATTAATTTTAAAAGTTGATAATCCT
56	32	TCAAAAGTTTTTTTAATAGCCCGGGCGCTGGC
57	45	TAAAGGGAGCGGAAGAACAGTTTGGAACAAGAGTCCCGGGCGAGA
58	49	AATTCATCAATAACCCTTCTGACCTTTTTTTTGAAAGCGTCAGTGAGGC
59	33	TATGGTTGCTTTGACGAGCCAGACAACCTGAGA
60	49	ACGGAATAAGTTACAAAGAACGCGTTTTTTTAGAAAACTTAAGTATTAG
61	39	CTAAAGCATGCAAATACCGAACGAACTAAATTATAGATT
62	32	CAAGAAATTTTTTTCAATGAAATTTGAGGCAG
63	33	CCGGAACCAGAGCCACCACTACCGAAGCCGCCA
64	44	AACGTAGAAGCGACATTCATTTTTTTACCGATTGAAGACTGTAG
65	49	CAACAGTAGGGCCGCAATAATAACTTTTTTTGGAATACCCAAATTCATA
66	51	TGGTTTACCAGCGCCACAATAGAAAAAGAACTGGCATGATTAAGTATTTTG
67	42	AAAACATCGACCGACCGTTTTTTTTGTGATAAATAACAACGC
68	51	CAACATGTAATTTAGGCCATATTTAAGGCGTTAAATAAGAATAATTAATTG
69	32	AAGCGGATTTTTTTTTGCATCAAGCACTCCAG
70	42	TAACAACCGGATGGGCAAACTCCAACAGAGCCCGAAGTATCG
71	31	GGTGGCATCCAAAGGCGTTGATTCCCCTTTA
72	31	CTAATTTACCTTTCCTTAAAGTAATTAAGAG
73	29	CCTGTGTATACGAGGTGCGGGCCTATAAA
74	29	CGGGTAAAAAACGATAGTTGCGCGAAGCA
75	30	CACCGAGTAAAAGAGTTTATAATAAGAATA
76	30	GTCAGACGATTGGCCTCAGGAGGAGCAATA
77	31	GTCACCAATCGACAGACGGAAATTAGGGTTT
78	42	GCCTCAGGCCCGTGGGAACAAACGGCGGCGACGACAAGACTT
79	29	GATAGGTGCATCTGTAATTCGAGTGCAAC
80	40	TGGGGCGCCGGTAATCGAAATGGTAGTTTGACCATTAGAT
81	51	TCAGGGAACAAACTACAACACTAAAGGACACCACCCCCTCAGAACCGCCAC
82	42	CTGTATGCAGCCCTGTTTCGTCACCAGTTAGCAAGCCGCCAC
83	51	AGAGGCGGAAAACAAAATTATTAAGACGTTACAAATCGCCTGATTGCTTTG
84	42	TGTAAATATTTGAAGAAACAAACATCAAAATTATTTCGGGAG
85	38	TCAAAAAAGTCAGAGTACCACAAGATTCATCAGTTGAG
86	40	GATTAGTTTCCCAATCCCCCGACTGAACGCGAGGCGTTTT
87	40	GAAAAATACAAGCAAGCCGCCTGTCAACATGTTCAGCTAA

88	38	AGCTGCAACAGCTGAGGAATCGTAGACTGGATAGCGTC
89	51	ACGTTGTCCCCGGGTACCGCACTGCCCGTTTTCCCGCGATTAAGTTGGGTA
90	42	CATAAAGGGTCATACGACTCTAGAGGATAAAACGAGAAAGGG
91	46	ATAACCCGGAACCGACAAGAACCGACGGTCTGTTACTTAGCCGGAA
92	51	GAGTAACAGTGAATAAGGCGGGAAGAAAGGGGTCAGAGTGTACTGGTAATA
93	42	TAATCATCAGAACGAAAGCTGCTCATTCAGTGCCCACATGGC
94	51	GGATCGTGGCTTTGAGGACTACAACGGAAGGCCGCGTCGCTGAGGCTTGCA
95	42	AAAAGAAAAGTTTCGCAACGGCTACAGACACCCTCCACGCAT
96	46	TTTAAATATCCAGATTTACATTGCGGCCTTGCCTGAGTAGAAGAAC
97	46	GACCAGTATCATCACCTACCATACAGAAGGTCATTTTGCGGAACAA
98	51	ACCATCATAAAGCACTAAAGCGCGTAACGGCCCACGCGAAAAACCGTCTAT
99	42	AAGGAAGCCCCGATGGGTCGAGGTGCCGCCCAAATCGTGGAC
100	46	AATGCGCAGGCCGAGTCCATCACGCGGGAGACGTGCTTTCCTCGTT
101	51	GAGAGCCCCCTCAATCAATCAACTCGTAAACAGTGGGCGGTCAGTATTAAC
102	42	AGAGCCGCAACAGTCCTCAAATATCAAAAGCAGCACAGAAGA
103	46	GCCATCTAGCCACCATATTCACAGAACCGCGCCTCCCTCAGAGCCG
104	40	ATTAGCAATTCGGTCATTTAGAGCGTCACCGACTTGAGCC
105	46	TATAACTTGGCAACGACAAAAGGAATACATACGCAGTATGTTAGCA
106	46	CAGATAGTACAAATGAGGCATTTAGTATCACATAATTACTAGAAAA
107	51	ATTTTTGCCGTCGGATTCTTTCTGGTGCTTTGATATTAGAGAGTACCTTTA
108	51	GGCGCATCGTAACCGTCACGTTGGCTTTCATCAACATGCTGAATTCAAAGC
109	54	AACAAGAGAATCGATGCTGAGAGGAAGTTTCATTCCATAGATTTCAATAACCTG
110	51	TGAGAATAGAAAGGAAAACAGTTGATAAGTGCCGTCGACCGCCACTCATTT
111	48	ATTCCACAGAGGATTTTTGATATAAGTATAGTTTAGTACCCAATAGGA
112	51	AACATAGCGATAGCTTCCTTAGAAAACAGAAATAAAGAACGGATATCGCGC
113	48	TAACAATTTCCGTCGCTGTAGATTTTCAGGTTTTTACACATTTCAATT
114	54	AAACAGCCATATTATTTTGCCAGCCTGAGAGAGTTGCATTCTAATGCGGGAGGT
115	54	TACCGCACTCATCGAGCGGGTATATATAAAGTACCGACAATAAATTATCAACAA
116	51	CATTAATTGCGTTGCGTGAGTGACATTCGCCATTCAGCTGCAAGAGTCACG
117	48	TCGTAATCATTGTAAAGAACTGTTGGGAAGGAGCTGGCCGGCCAGTGC
118	51	TATACCAGTCAGGACGTTAAGAAAAGCCAGAATGGAAGATACAGGTGCCTT
119	33	ATTTCGGAACCTATAGTAAGCGTCATGTATAAA
120	51	ACCAAGCGCGAAACAAGACCCCCAGCTTGCTTTCGAGATATTCGTTTTGCG
121	48	TTTCATGAGGTACACTATCTTAAACAGCTTGCATCGCCAGCAGCGAAA
122	51	AAGTGTAGCGGTCACGGCGCTAGAGATAGGGTTGAGTTCAAAGGTACGTGA
123	51	ACTTTACAAACAATTCGATTTAGTTTCAAATATATTTGAGGTGACCACGCT
124	48	GTTGGCAAATTCAATAGTTTCATCTTCTGACCCACCAGAATGAAAAAT
125	54	CGCGTTTTCATCGGCATAGCGTCGGGAGGGAAGGTAATATCACCCAGCAAAATC
126	51	CCAGCTTTCCGGCACCGAAGATCAAAGATTAAGAGGAGTCAGGAAGAGGTC
127	42	ATTTAGGAAGGTAATTGAGTTTTTTTCGCTAATTAGAAACCA
128	32	GTTAAGCCCAATAATCAACCCACAAGAATTGA
129	42	CCTCAGAGCATTGCGAATATTTTTTTATAATTTCTAATAGTA
130	33	CAGTTAAACGTAACAGTAGTAAATTGGGCTTGA
131	47	GATGGTTTAATTTTCTGAAACATGTTTTTTTAAAGTATTCGATAGCA
132	32	GGCTCCAAAAGGAGCAAAATCTCCAAAAAAAA
133	49	CGAGGCGCAGGATATTCATTTTTTTTTACCCAAATCATGCCCCCTGCCT

134	32	GATTAGGATTAGCGTTGACTCCTCAAGAGAAG
135	33	TGGGCGCGCATTAATGAATCGGCCAACGCGCGG
136	47	GGAGAGGCGGTTTCAGAAAACGAGTTTTTTTAATGACCAATGCCACT
137	49	ACGCCAGGGCTTTCCAGTCTTTTTTTGGGAAACCTGTTGAATCCCCCTC
138	32	GACTATTATAGTCACGATCAGGTCTTTACCCT
139	42	TGCAGAACGCGTTTTTATTTTTTTTTTTCATCGATCCTGAAT
140	40	AGCAAATCATGCCCCAGTTTTTTTCAGGCGATTATCCGCT
141	33	AGTGTTTCTTTAAAGGGATTTTAGACAGGAACG
142	47	GTACGCCAGAATTATTTTTGAATGTTTTTTTGCTATTAATCTAAAAT
143	53	ACCCAGCTAAGCGTCTGGTCCACGCTGGTTGATATAGACCGCGCCCAATAGCA
144	32	TCGGCAAAATCCCTTCGATGGTGGTTCCGAAA
145	40	CGTGGCAACGTATACTAAACAGGCGCTACAGGGCGCGTAC
146	51	AATTGAGACAACTATAATGGTTTGAAATCCATTAAGCGAACTGATAGCCCT
147	42	AATACCAAGCTGAGAAGAGTTTTTTTTCAATAGAAGTTTTGT
148	33	TCACAATAAATATAAAAGAAACGCAAAGACACC
149	32	AGACTACCTTTTTACAAAATCATAGGTCTGAG
150	52	CAAATCCAATCGCAAGACTCCTTATTACATAAAGGATATGTAAATGCTGATG
151	42	ACATTTCGCTAAAACTAGCTTTTTTTATGTCAAGTACATAAA
152	33	AAATACCTAACAATATTACCGCCAGCCATTGCA
153	28	ATTTTTTTGTTAGAGCTTGACGGGGAAA
154	47	GCCGGCGAACGTTCTGGCCTTCCTTTTTTTTGTAGCCAGTGTAGATG
155	32	AAAGCCCCAAAAACATCCGGTTGATAATCAGA
156	52	TGTTAAAATTCGCAATAACATCACTTGCTGGTAATTGTAAACGTTAATATTT
157	52	TCAAAAATAATTCGACTATTAAAGAACAAGTTTTTAACCAATAGGAACGCCA
158	32	ACATGTTTTAAATACTATAATGCTGTAGCTCA
159	33	GAAGCGCCAATGAAAATAGCAGCCTTTACAGAG
160	33	CAGGCGCTGAACTGACCAACTTTGAAAGAGGAC
161	49	AGCGAACCTAAATAAGAAATTTTTTTCGATTTTTTGTATACATAACGCC
162	52	ATAAAAACCAAAATCGACCTGCTCCAAATCATAAGTCGTTTACCAGACGACG
163	33	GATTGTTAATATTCCTGATTATCAGATGATGGC
164	52	GGCCAACAGAGATATGAGTAACATTAAGCGGAATTAATAAAAGGGACATTCT
165	33	GCATTGATGACCCTCAGAGCCGCCACCAGAACC
166	33	AGAATCGCATCTTACCAGTATAAAGCCAACGCT
167	40	GCTATCTCGGAACCCACCCTCAGTTTCATAATCAAAATCA
168	52	AAGGAAACCGAGGAAAACACCGGAATTATGCGTTACCGAACAAAGTTACCAG

Supplementary References

1. Cagan,J. and Mitchell,W.J. (1993) Optimally Directed Shape Generation by Shape Annealing. *Environ Plann B Plann Des*, **20**, 5–12.

2. Kirkpatrick,S., Gelatt,C.D. and Vecchi,M.P. (1983) Optimization by Simulated Annealing. *Science*, **220**, 671–680.

3. Stiny,G. (1980) Introduction to Shape and Shape Grammars. *Environ Plann B Plann Des*, **7**, 343–351.

4. Metropolis,N., Rosenbluth,A.W., Rosenbluth,M.N., Teller,A.H. and Teller,E. (1953) Equation of state calculations by fast computing machines. *The journal of chemical physics*, **21**, 1087–1092.

5. White,S.R. (1984) Concepts of scale in simulated annealing. *AIP Conference Proceedings*, **122**, 261–270.

6. Nourani,Y. and Andresen,B. (1998) A comparison of simulated annealing cooling strategies. *J. Phys. A: Math. Gen.*, **31**, 8373.

7. Suppapitnarm,A., Seffen,K.A., Parks,G.T. and CLARKSON,P.J. (2000) A Simulated Annealing Algorithm for Multiobjective Optimization. *Engineering Optimization*, **33**, 59–85.

8. Suppapitnarm,A., Parks,G.T., Shea,K. and Clarkson,P.J. (2004) Conceptual design of bicycle frames by multiobjective shape annealing. *Engineering Optimization*, **36**, 165–188.

9. Huang,M., Romeo,F. and Sangiovanni-Vincentelli,A. (1986) An efficient general cooling schedule for simulated annealing. In *Proc. IEEE Int. Conf. on CAD (ICCAD 86)*.

10. Triki,E., Collette,Y. and Siarry,P. (2005) A theoretical study on the behavior of simulated annealing leading to a new cooling schedule. *European Journal of Operational Research*, **166**, 77–92.

11. Edelsbrunner,H. and Mücke,E.P. (1994) Three-dimensional alpha shapes. *ACM Trans. Graph.*, **13**, 43–72.

12. Bohlin,J., Matthies,M., Poppleton,E., Procyk,J., Mallya,A., Yan,H. and Šulc,P. (2022) Design and simulation of DNA, RNA and hybrid protein–nucleic acid nanostructures with oxView. *Nat Protoc*, **17**, 1762–1788.

13. Poppleton,E., Bohlin,J., Matthies,M., Sharma,S., Zhang,F. and Šulc,P. (2020) Design, optimization and analysis of large DNA and RNA nanostructures through interactive visualization, editing and molecular simulation. *Nucleic Acids Res*, **48**, e72.

14. Poppleton,E., Romero,R., Mallya,A., Rovigatti,L. and Šulc,P. (2021) OxDNA.org: a public webserver for coarse-grained simulations of DNA and RNA nanostructures. *Nucleic Acids Research*, **49**, W491–W498.

15. Sengar,A., Ouldridge,T.E., Henrich,O., Rovigatti,L. and Šulc,P. (2021) A Primer on the oxDNA Model of DNA: When to Use it, How to Simulate it and How to Interpret the Results. *Front Mol Biosci*, **8**, 693710.

16. Riquelme,N., Von Lücken,C. and Baran,B. (2015) Performance metrics in multi-objective optimization. In *2015 Latin American Computing Conference (CLEI)*.pp. 1–11.

17. Nečas,D. and Klapetek,P. (2012) Gwyddion: an open-source software for SPM data analysis. *Central European Journal of Physics*, **10**, 181–188.
